# Natural Language Response Formats for Assessing Depression and Worry With Large Language Models: A Sequential Evaluation With Model Pre-Registration

**DOI:** 10.1177/10731911251364022

**Published:** 2025-09-20

**Authors:** Zhuojun Gu, Katarina Kjell, H. Andrew Schwartz, Oscar Kjell

**Affiliations:** 1 Lund University, Skåne, Sweden; 2 Stony Brook University, USA

**Keywords:** artificial intelligence, large language models, natural language, natural language processing, psychological assessment, depression, anxiety

## Abstract

Large language models can transform individuals’ mental health descriptions into scores that correlate with rating scales approaching theoretical upper limits. However, such analyses have combined word- and text responses with little known about their differences. We develop response formats ranging from closed-ended to open-ended: (a) select words from lists, write (b) descriptive words, (c) phrases, or (d) texts. Participants answered questions about their depression/worry using the response formats and related rating scales. Language responses were transformed into word embeddings and trained to rating scales. We compare the validity (concurrent, incremental, face, discriminant, and external validity) and reliability (prospective sample and test–retest reliability) of the response formats. Using the *Sequential Evaluation with Model Pre-Registration* design, machine-learning models were trained on a development dataset (*N* = 963), and then *pre-registered* before tested on a prospective sample (*N* = 145). The pre-registered models demonstrate strong validity and reliability, yielding high accuracy in the prospective sample (*r* *=* .60–.79). Additionally, the models demonstrated external validity to self-reported sick-leave/healthcare visits, where the text-format yielded the strongest correlations (being higher/equal to rating scales for 9 of 12 cases). The overall high validity and reliability across formats suggest the possibility of choosing formats according to clinical needs.

Recent advances in artificial intelligence, including natural language processing (NLP) and deep learning, have substantially improved the analysis of textual data (Bommasani et al., 2021; Vaswani et al., 2017) and the ability to assess psychological aspects of language (e.g., Boyd & Schwartz, 2021; Demszky et al., 2023; Kjell et al., 2024). AI-based language analyses have successfully been used in research for psychological assessments of, for example, personality (converging with rating scales; Cutler, & Condon, 2023; Kwantes et al., 2016; Schwartz et al., 2013), depression (from medical records, Eichstaedt et al., 2018, and Vaci et al., 2020; assessed with rating scales in Matero et al., 2021), and anxiety (assessed with rating scales, Rutowski et al., 2020). The types of language that have been used for computational language assessments have ranged from social media texts (e.g., from Facebook [Marengo et al., 2021; Park et al., 2015] and Twitter [Coppersmith et al., 2014; Eichstaedt et al., 2015]), audio recordings of everyday conversations (Gumus et al., 2023; Sun et al., 2020), and open-ended responses to prompted questions (Kjell et al., 2019). The latter approach recently yielded the highest accuracy in converging with rating scales, where respondents described their state of mind with descriptive words and text responses (Kjell et al., 2022). Combining responses of different formats (words and text responses) across different construct questions (responses about harmony in life and satisfaction with life) was used to assess well-being rating scales with an accuracy (*r* = .85, *N* = 608) approaching the rating scales’ own reliability (*r* = .71–.84, *N* = 608), which is considered a theoretical upper limit of assessment accuracy (Kjell et al., 2022).

The overall aim of this study is to develop and evaluate open-ended response formats for assessing mental states, with a focus on enhancing clinical utility. Mental health assessments are critical for effective diagnosis, treatment planning, and monitoring progress (Hunsley & Mash, 2007; Shen et al., 2018; Stahnke, 2021). Current methods, such as rating scales, are limited in their ability to comprehensively capture the complexity of patients’ unique subjective experiences (Fried, 2017; Kjell et al., 2024), which can lead to missed nuances and suboptimal treatment outcomes. To address these challenges, this study develops and evaluates different response formats ranging from closed to more open, including (a) selecting descriptive words from pre-defined word lists (the select words format) and *writing* their own, (b) descriptive words (the write words format), (c) descriptive phrases (the write phrases format), or (d) descriptive texts (the write texts format).

The focus is on the two most common mental health symptom domains measured by rating scales: depression and anxiety/worry, where self-reported subjective and behavioral experiences (e.g., low mood, worry, sleep disturbances) are key symptoms. Our selection of response formats offers varying levels of openness to describe relevant experiences, enabling a tailored approach to assessment that can potentially balance the need for brevity with the richness of open-ended responses. By exploring these formats, this study aims to provide clinicians and researchers with tools that can achieve high accuracy and reliability as well as offer more comprehensive insights into patients’ mental health experiences.

## Current Clinical Assessment Practices and Limitations

Current clinical practices primarily rely on a combination of closed-ended rating scales and unstructured clinical interviews to assess mental health problems. In an international survey of clinicians from the United States (*N* = 148), the United Kingdom (*N* = 162), the Netherlands (*N* = 105), and Sweden (*N* = 127), revealed that clinicians working with mental health assessments primarily use unstructured clinical interviews (83%) and rating scales (83%), followed by semi-structured interviews (65%) and structured interviews (40%; Navandi et al., in preparation). The survey also highlighted that mental health assessments require a significant investment of clinicians’ time, with a median reported assessment time of 60 min, underscoring the time-intensive nature of current practices.

Unstructured interviews allow clinicians to explore patients’ mental health in a more open-ended and conversational manner. However, these interviews are often time-intensive, require significant clinical expertise, and their reliability can vary widely (Regier et al., 2013). Semi-structured and structured interviews offer greater standardization and reliability, but they remain time-consuming and may still rely on the clinician’s interpretation for nuanced responses (Hoffman et al., 2024; Lundqvist et al., 2022). The validity of clinical judgment is also compromised by cognitive biases and factors that increase susceptibility to errors, such as clinician fatigue (Bowes et al., 2020; Saposnik et al., 2016).

The quantification of psychological assessments has typically been achieved using closed-ended rating scales—based on questions or statements coupled with a limited set of pre-defined, categorical, or rating scale-based response formats (e.g., Likert, 1932). The closed-ended format provides consistency through predefined questions and response options, which are easy to administer. However, the closed-ended format of rating scales inherently limits the comprehensiveness and nuance of the information they can capture, often reducing patients’ complex mental health experiences into closed-ended, forced-choice responses (Kjell et al., 2024). This closed-ended format restricts respondents from fully expressing their unique symptoms and experiences (Kjell et al., 2019; Pennebaker & Beall, 1986). Moreover, the closed-ended format comes with the risk that the scale developers potentially miss important aspects to capture a diagnosis or psychological construct (Fried et al., 2017; Kjell, 2011).

These limitations emphasize the need for more open-ended assessment methods that are also reliable in capturing the subjective and multifaceted nature of mental health problems. Open-ended language-based assessments offer a promising alternative, leveraging natural language responses to enable individuals to describe their unique experiences while producing reliable measurements with high accuracy.

## Natural Language as the Foundation of Psychological Assessments

Language is at the core of the communication between patients and clinicians in clinical settings, playing a key role in assessment, treatment as well as evaluation of progress (Cruz & Pincus, 2002; Greenberg & Pascual-Leone, 2006; Levinson & Holler, 2014). An open-ended approach using natural language as a foundation for quantitatively assessing mental states has many potential benefits. First, natural language is the natural way of communicating complex psychological states of mind (e.g., Tausczik & Pennebaker, 2010). Second, natural language comprises great measurement characteristics, including high range, resolution, dimensionality, and openness (Kjell et al., 2024). The range in language facilitates the description of extremes (e.g., *despondent* and *euphoric*). The resolution in language enables descriptions of detailed nuances (e.g., *despondent*, *sad*, *happy*, *euphoric*). The multi-dimensionality of language facilitates capturing complex states that vary in more than one dimension (e.g., both *valence* and *arousal*). The openness of language affords the option to create personal responses, which is very difficult to capture exhaustively with predefined alternatives.

Third, the openness of language can enhance the content validity of mental health assessments by capturing a broader and more comprehensive representation of clinical constructs (e.g., depressive disorders). In contrast, the omission of important response alternatives in closed-ended methods can undermine construct validity by failing to account for the full breadth and complexity of the targeted disorder. For example, *mental pain* is commonly reported as a significant aspect of depression. To identify symptoms that matter most to patients, Chevance et al. (2020) asked participants to describe “the most difficult aspect of depression to live with or endure.” They found that “mental pain” was the most frequently mentioned symptom. Yet, it is notably absent from the DSM-5 criteria and the closed-ended rating scales commonly used to assess depression, including those in this study. In previous studies using language-based assessments, descriptive words such as “painful” emerged as significantly related to high depression (Kjell et al., 2021). This highlights the limitations of predefined response categories and the potential of open-ended responses in capturing the comprehensive spectrum of patients’ experiences.

Fourth, language-based responses also carry more information than closed-ended responses. Based on the information theory (Shannon, 1948), research has shown that natural language-based responses carry 4.8 times more information than closed-ended rating scales (Kjell et al., 2024), offering richer data. These favorable measurement characteristics indicate that using language-based responses has the potential to improve mental health assessments. This study focuses on comparing the characteristics of different response formats.

Different response formats may differ in multiple relevant dimensions for assessment, such as the type and amount of information they capture, the assessment accuracy they produce, the way responses can be visualized, and the time taken for patients to complete them. Additionally, asking respondents to answer the same question using different response formats may encourage more thorough answers, as each format may elicit different perspectives and capture unique information. Such an approach aligns with the cognitive interview theory (Willis, 2004), which emphasizes how different questions can activate diverse cognitive processes, leading to the retrieval and articulation of different facets of an individual’s experience, resulting in a more comprehensive answer. Hence, the response formats might complement each other, potentially enhancing the overall accuracy of a mental health assessment when combined (i.e., incremental validity).

## Computational Language Assessments

Computational language assessments based on individuals’ naturally occurring language, such as their Facebook statuses and tweets, have been analyzed with AI to assess psychological constructs. For example, Facebook statuses have been analyzed to assess individuals’ Big Five personality factors (*r* = .54–.63, *N* = 1,943 in Lynn et al., 2020; *r* = .30–.46, *N* = 4,824 in Park et al., 2015; *r* = .28–.42, *N* = 71,000–75,000 in Schwartz et al., 2013), depression levels (*r* = .39, *N* = 1,000; Schwartz et al., 2014), and well-being (*r* = .20–.30, *N* = 2,198; Schwartz et al., 2016; see also Kjell et al., 2024). Facebook statuses have also been used to predict depression diagnosis from medical records with an accuracy of AUC = 0.72 (*N* = 683; Eichstaedt et al., 2018), where an *AUC* of .70 is considered the threshold for “sufficient” discrimination (Mokkink et al., 2018, p. 29). However, these social media analyses rely on language that has not been directly prompted by specific questions, which is a key difference from this study.

Probed-based computational language assessments, where respondents describe their answers using natural language that is analyzed with AI, have shown promise in assessing psychological constructs with high validity. Computational language assessments where respondents describe their depression, worry, harmony in life, and satisfaction with life using descriptive words have been demonstrated to *measure* the degree of the constructs (i.e., concurrent validity; Kjell et al., 2019). Computational language assessments have further been shown to *describe* and *differentiate* well between similar constructs; for example, statistically significant words describe and differentiate well between depression versus worry, where, when compared with each other, depression is related with theoretically relevant words such as *sad*, *worthless*, and *lonely*, and worry with words such as *anxious*, *nervous*, and *scared*. Hence, these analyses have shown that language-based assessments have high face validity; here, we will examine how well the different response formats describe the different constructs and their face validity.

### The Response Format

To date, respondents have been asked to answer the questions using descriptive words or descriptive text (Kjell et al., 2019). Initially, the answers were analyzed using a bag-of-words approach called Latent Semantic Analyses (Landauer & Dumais, 1997)—where words are represented with a numeric representation that does not take the order and context of a word into account. This bag-of-words technique produced considerably more accurate results with the descriptive words (*r* = .34–.72, *N* = 91-852) than with the descriptive text format (*r* = .19–.49, *N* = 92-689; Kjell et al., 2019). A re-analysis of the data, with a *large language model* (based on a technique called *transformers*; Vaswani et al., 2017)—that takes words’ order and context into account—has produced high accuracy for both descriptive words (*r* = .66–.79, *N* = 608; Kjell et al., 2022) and descriptive texts (*r* = .61–.74, *N* = 608; Kjell et al., 2022). The research has also demonstrated that combining the two response formats for two different constructs produces even higher accuracies (incremental validity; *r* = .85, *p* < .001, *N* = 608) that rival the rating scales’ own reliability as measured with test–retest correlation, corrected item–total correlation average, and inter-item correlation average (*r* = .71–.84, *N* = 608). This accuracy is an essential achievement because the scale’s own reliability can be seen as a theoretical upper limit of predicting it. Hence, the large language models have opened up the possibility of using more complex response formats while attaining high measurement accuracy.

## Large Language Models in NLP

Large language models are now the foundation for most state-of-the-art AI-language systems and have been described as a “paradigm shift” (Bommasani et al., 2021, p. 1). Large language models can efficiently represent a word’s meaning based on the context in which they were used. The context a word is in *plays* a vital role in determining its meaning. For example, consider how the context changes the meaning of the word *play* in the previous sentence, as compared with “I *play* my guitar” versus “I watched a *play.*” This technique has led to increased accuracy in standard NLP tasks such as grammatical acceptability (Warstadt et al., 2019) and question answering (Rajpurkar et al., 2018). The ability of large language models to more accurately represent language may provide more options for using different response formats. Whereas previous computational language assessments have found the highest assessment accuracy of the write words response format, the advent of large language models that more accurately account for grammar and syntax, including word order, may provide even higher accuracy than those previously achieved.

## Depression and Anxiety

Here, we focus on assessing depression and anxiety for three primary reasons. First, depression and anxiety are highly prevalent in the general population, representing two of the most common mental health issues globally (Baxter et al., 2013; Lépine & Briley, 2011). Given their high prevalence, improving assessment methods for these issues has significant utility. Second, the assessment of depression and anxiety heavily relies on subjective experiences, as these disorders are characterized by internal states such as low mood and worry (e.g., see the Diagnostic and Statistical Manual of Mental Disorders, Fifth Edition, DSM-5; American Psychiatric Association, 2013). Open-ended language-based assessments provide an opportunity to more comprehensively quantify these subjective experiences. Last, previous research using language-based assessments shows that writing words to describe one’s level of depression and anxiety significantly converges with the individual item scores and total scores of corresponding rating scales (Kjell et al., 2021). In this study, we build on these insights to examine whether different response formats can further improve the assessment validity and reliability of depression and anxiety measures.

## Research Questions and Hypotheses

We evaluate the four response formats: (a) the select words, (b) the write words, (c) the write phrases, and (d) the write texts response format according to their:

### Validity of Models During Development

- *Concurrent (criterion) validity.* How well do they converge with rating scale scores?
- *Concurrent (criterion) validity across sample sizes.* To what extent does the model accuracy increase with larger training sample sizes?
- *Incremental validity.* How well do combinations of response formats converge incrementally with rating scales?
- *Face validity.* Is the language that is predictive of depression versus anxiety face valid (when depicted in word visualizations)?
- *Discriminant validity.* How well do they differentiate between depression and anxiety?

### Validity and Reliability of Pre-Registered Models

vi) *Prospective sample reliability.* How accurate (convergence with rating scales) are pre-registered models on data from a new set of participants?
This part involved two pre-registered hypotheses stating that the pre-registered models of depression and anxiety/worry yield scores that:
  a) are positively associated with corresponding rating scale scores, and
  b) achieve at least a moderate correlation *r* > .50 for total scores where depression language predicts depression rating scales and worry language predicts worry/anxiety rating scale scores (note that the 99% confidence interval for the lowest correlation in the training set [*r* = .59] is above .50 0[.53–0.64 *N* = 963]).

vii) *Test–retest reliability in a prospective sample.* How high is the 2-week test–retest correlation for the pre-registered models?
viii) *External validity in a prospective sample.* How well do the pre-registered models’ predictions correlate with a few self-reported behaviors associated with depression and anxiety (e.g., sick leave due to mental health issues)?

### Descriptive Characteristics of the Response Formats

ix) *Information content*. How much information content is captured by each response format?
x) *Time burden*. What is the response time for each format?

## Methodological Disclosure and Data Accessibility Statement

We report how we determined our sample size, all data exclusions, all manipulations, and all measures in the study. We make all the research materials, and the anonymized prospective data, publicly available anonymously at the OSF (https://osf.io/syren/?view_only=9eebf3e448b447ae89dd288d3107cb03).

## Methods

We use the *Sequential Evaluation with Model Pre-Registration* (SEMP; Kjell et al., 2025) study design, which aspires to adhere to good scientific practices (i.e., pre-registration) combined with development practices for achieving robust AI model evaluation (e.g., out-of-sample testing). Here, SEMP involves registering models developed in a *development set* (*N* = 963) and pre-registering hypotheses before testing them on a new *prospective participant set* (*N* = 150).

### Participants

#### The Development Data

Participants were recruited online using Prolific (Palan & Schitter, 2018) in August 2020. A mixture of unscreened and screened respondents were recruited. Screened participants reported being diagnosed with an ongoing mental health diagnosis of Major Depressive Disorder (MDD) or Generalized Anxiety Disorder (GAD). Overall, 258 participants reported having been diagnosed with MDD, 259 with GAD, and 491 were recruited from an unscreened population. Forty-five participants were excluded for not answering the attention check items correctly (*N* = 32) or not having any data saved (*N* = 13). Out of the remaining 963 participants, 571 identified as female, 388 as male, two preferred not to say, and two did not respond. The average age was 33.3 (*SD* = 11.2; *range* = 18–77) years. Participants reported being from the United States (*n* = 284) and the United Kingdom (*n* = 679), and 903 reported English, 16 reported European languages, 16 reported Asian languages, 1 reported Russian, 3 reported African languages, and 1 reported Esperanto as their first languages, with 23 participants not responding^1^. The training dataset sample size was determined based on previous studies (Kjell et al., 2019, 2016). In the training set, 7 participants had missing data in the write text response format questions (7 missing for the depression and 3 missing for the worry questions).

#### The Prospective Test Data

Participants (*N* = 150) in the prospective test data were also collected from Prolific in January 2024 (i.e., collected 41 months after the development set). The size was based on finding reliable estimates of the correlations that we were interested in from analyses of the training dataset. Five participants were excluded from the analyses: three did not pass the attention check items, and two did not respond to the write text questions of depression and anxiety.^2^ The rest of the participants (*N* = 145) were further invited to take part in a longitudinal study with 2 weeks between Time 1 and 2 (T1, T2). At T1, participants had a mean age of 41.6 (*SD* = 12.6, *range* = 19–81) years; 101 female, 43 male, and 1 other. Their reported nationality included 126 from the United Kingdom, 5 from the United States, and 14 from other countries. At T2, 122 (84.1%) participants returned to answer the same survey again; they have an average age of 42.3 (*SD* = 12.6; *range* = 19–81) years, and 87 identified as female, 34 as male, and 1 as other. All participants in the prospective test data have English as their first language.

### Measures

#### Screening Question

The participants were asked to report whether they had any previous or ongoing diagnosis of MDD, GAD, or other mental health problems (see Supplement Appendix (SA) 1 for details).

#### The Response Format Questions

The open-ended response format for descriptive words (developed by Kjell et al., 2019) was used and adapted for the different response formats. Previous research piloted different question prompts and instructions, finding that this format with a question prompt coupled with instructions to elaborate using open-ended responses works very well (Kjell et al., 2019). An example question of the write phrases and write text formats is exemplified below:

**Over the last 2 weeks, have you been depressed or not?**

Please answer the question by typing 5 descriptive words or phrases below that indicate whether you have been depressed or not. For example, if you have been depressed, then write more and stronger words or phrases describing this, and if you have not been depressed, then write more and stronger words or phrases describing that.

Write descriptive words or phrases relating to those aspects that are most important and meaningful to you.

Write one to five words in each box.

**Over the last 2 weeks, have you been depressed or not?**

Please answer the question by typing at least a paragraph below that indicates whether you have been depressed or not. Try to weigh the strength and the number of aspects that describe if you have been depressed or not so that they reflect your overall personal state of depression. For example, if you have been depressed, then write more about aspects describing this, and if you have not been depressed, then write more about aspects describing that.

Write about those aspects that are most important and meaningful to you.

Write at least one paragraph in the box.

The question was adapted for worry (“worried or not”), and the instructions were adapted for the select words (“answer the question by selecting 5 descriptive words”), and the write word formats (“answer the question by typing 5 descriptive words”; see SA2). The list of words in the select words format, comprised the 31 most frequent words participants used to describe their depression (e.g., *blue*, *lonely*, *tired, hopeful*, *happy*) or worry (e.g., *anxious*, *nervous*, *worried*, *calm*, *peaceful*) in previous research (Kjell et al., 2019; see SA3 for the lists).

#### Rating Scales

Several commonly used clinical scales are used to measure depression and anxiety severity, with the aim for the scales to complement each other. The Patient Health Questionnaire-9 for depression (PHQ-9, Spitzer et al., 1999) and the GAD-7 for anxiety (GAD-7, Spitzer et al., 2006) are closely aligned with the symptoms defined in the DSM manual for MDD and GAD, respectively. The Center for Epidemiologic Studies Depression Scale (CES-D, Radloff, 1977) complements the PHQ-9 by including some depression-related symptoms not included in the DSM, such as indirectly related interpersonal issues. The Penn State Worry Questionnaire (PSWQ, Meyer et al., 1990) complements the GAD-7 by focusing on experiences of worry.

##### The PHQ-9

The PHQ-9 (Spitzer et al., 1999) measures MDD as defined in the DSM-IV. The rating scale comprises nine items addressing symptoms such as “little interest or pleasure in doing things.” The scale uses a four-point scale (0 = *not at all* to 3 = *nearly every day*). The items refer to experiences from the last 2 weeks. In the current study, Cronbach’s alpha was .95, and MacDonald’s hierarchical omega was .84.

##### The CES-D

The CES-D measures depressive symptoms severity (Radloff, 1977). It includes 20 items referring to the last week, such as “I could not get ‘going,’” coupled with a four-point scale (0 = *rarely or none of the time [less than 1* *day]* to 3 = *most or all of the time [10–14* *days]*). Cronbach’s alpha was .96, and MacDonald’s hierarchical omega was .91 in this study.

##### The GAD-7

The GAD-7 measures GAD as described in the DSM-IV (Spitzer et al., 2006). It comprises seven items, for example, “Feeling nervous, anxious or on edge,” referring to the last 2 weeks, coupled with a four-point Likert scale (0 = *not at all*, to 3 = *nearly every day*). Cronbach’s alpha was .93, and MacDonald’s hierarchical omega was .87 in this study.

##### The PSWQ

The PSWQ was developed to assess worry/anxiety symptoms severity (Meyer et al., 1990). The rating scale comprises 16 items, such as “My worries overwhelm me.” The scale uses a 5-point Likert scale (0 = *not at all typical of me*, 4 = *very typical of me*). In this study, Cronbach’s alpha was .91, and MacDonald’s hierarchical omega was .89.

#### Demographics and Mental Health-Related Sick Leave and Healthcare Visits

The survey included asking participants to report their gender (including *Female, Male*, or *Other*), age, sick leaves (due to mental health issues) in the last 3 months and the last year, and healthcare visits (due to mental health issues) in the last year.

#### Attention Check Items

Attention check items instructing participants to select a specific rating scale response option (see SA4 for details) were included within the PHQ-9 and the GAD-7.

#### Procedure

All participants were informed about the nature of the study, that their participation was voluntary, that they could withdraw at any time without having to give a reason, and that their answers were anonymous. All participants provided their consent to take part and that their anonymized data could be shared openly. Then, participants are asked whether they previously had been diagnosed with MDD, GAD, or any other mental health problems. After that, the survey had five parts: First, participants answered the three open-ended response formats (i.e., write words, phrases, and texts), which were presented in random order across participants. Second, they answered the select words questions. Third, they filled out the rating scales: the PHQ-9, the CES-D, the GAD-7, and the PSWQ, which were randomized. Last, they reported the demographics and mental health-related sick leave and healthcare visits. Participants completed the study in a median time of 20.1 (mean = 24.0, *SD* = 12.8)min.

#### Analytical Methods

The analyses were conducted in R (R Core Team, 2020) and the text-package (version 1.2.1; Kjell, Giorgi, & Schwartz, 2023). The text-package enables social scientists to access open large language models, and machine learning and NLP methods. We employed large language models to convert language responses into word embeddings (i.e., numerical representation). These embeddings were then used as predictors in training regression-based machine-learning techniques to predict scale scores.

#### Pre-Trained Word Embeddings

We use a large language model called RoBERTa-large (Liu et al., 2019), which has been developed from text from Wikipedia and books. The RoBERTa-Large model has demonstrated strong alignment between language-based assessments and a range of psychological constructs, incorporating both self-reports and expert evaluations (Ganesan et al., 2021; Matero et al., 2024; Varadarajan et al., 2024). We did not pre-process the text data; however, RoBERTa-large automatically tokenises text using the Byte-Pair Encoding (Sennrich, 2015). RoBERTa-large represents each word (token) across 24 layers, each with 1024 dimensions. Only the second-to-last layer is used here, as empirical research shows that these work well for representing the meaning of words when modeling human-level language tasks such as assessing mental health issues (Ganesan et al., 2021). Word embeddings are aggregated to represent several words/text using the means (the default in the text-package).

#### Predictive Modeling Using Word Embeddings

The word embedding dimensions of the responses are used as predictors in ridge regression (Hoerl & Kennard, 1970) to predict the rating scale scores. Although there are many types of predictive models from statistical learning where embeddings can be applied, recent empirical studies have consistently shown that ridge regression achieves state-of-the-art results when using contextual embeddings (Ganesan et al., 2021; Matero et al., 2021; Kjell et al., 2022;). Tenfold cross-validation was used for model training. The dataset was split into a training set (90%) and a test set (10%). Within the training set, an analysis set was used for developing models with various penalties, and an assessment set was utilized to evaluate these penalties and select the optimal model for the testing set (for details, see Kjell, Giorgi, & Schwartz, 2023). The search grid in the ridge regression covered the range of 10^-16^–10^16^ with increases of times 10. The training sets were stratified into groups based on the outcome (y) using 4 bins. Pearson correlation between the observed and language-assessed scores is used to assess the accuracy. Appendix SA5 lists additional R packages involved.

#### Significance Testing the Assessment Accuracy of Models

To compare the errors of two prediction models, we compute the error for each prediction (i.e., *y*− $\hat{y}$ ), and then use a paired sample t-test to examine whether the errors significantly differ.

#### Standardized Difference Scores

To assess a form of discriminant validity of the different response formats, we evaluate their ability to converge with difference scores between rating scales. This is done by training word embeddings to evaluate the difference scores. The difference scores are computed by subtracting one normalized score from another (where the normalization includes subtracting the mean and dividing by the standard deviation). For example, a positive score from taking the normalized PHQ-9 score minus the normalized GAD-7 score, indicates greater depressive symptoms *relative to* anxiety symptoms. This method allows us to determine how well the embeddings capture the distinctions (the relative difference) between these related constructs.

#### Diversity Index

To quantify the information in responses, we used Shannon Entropy (Shannon, 1948). This measure is important in machine learning, as it indicates how much information the algorithms have at their disposal to learn from. In this article, we use the Diversity index, which quantifies the entropy or diversity in a set of values or probabilistic events, which is here the possible responses on a rating scale or a word-based response). Mathematically, the Diversity Index is computed as 2^entropy^, where *entropy* is calculated as the Shannon entropy:

H = − ∑*x* p(*x*) log(p(*x*)), where p is its probability mass function, and *x* is a set of values or probabilistic events.

#### The Magnitude of the Correlations

All correlations were computed using Pearson’s correlation. Correlations of .20 to .39 were interpreted as weak, .40 to .59 as moderate, .60 to .79 as strong, and above .80 as very strong (Kjell et al., 2022).

## Results

### Descriptives

Table 1 presents the correlations among rating scales and their mean, median, and standard deviation. The correlations between the rating scales range from *r* = .64–.91. The strong correlations indicate the convergent validity among the scales measuring depression and anxiety; notable though, the GAD-7 correlates stronger with the depression scales (*r* = .82–.85) than to the PSWQ (*r* = .64–.71).

**Table 1:** Pearson Correlations and Descriptives of the Rating Scales.

Measure	1	2	3	4	5	6	Mean	Median	*SD*
The development data (*N* = 963)									
1. PHQ-9	—	—	—	—	—	—	11.56	11	7.56
2. CES-D	.91	—	—	—	—	—	26.84	28	14.95
3. GAD-7	.82	.85	—	—	—	—	10.10	10	6.27
4. PSWQ	.64	.71	.76	—	—	—	42.17	45	15.53
5. Sick leave due to mental health last 3 months	.23	.24	.24	.15	—	—	4.38	0	17.05
6. Sick leave due to mental health last year	.23	.24	.23	.16	.90	—	16.47	0	65.05
7. Healthcare visits due to mental health last year	.25	.26	.21	.16	.25	.23	.96	0	2.64
The prospective data (*N* = 145)									
1. PHQ-9	—	—	—	—	—	—	8.25	7	7.09
2. CES-D	.92	—	—	—	—	—	20.16	18	14.63
3. GAD-7	.82	.86	—	—	—	—	7.84	7	6.43
4. PSWQ	.65	.70	.76	—	—	—	37.88	41	17.33
5. Sick-leave due to mental health last 3 months	.29	.30	.21	.15	—	—	2.49	0	11.97
6. Sick leave due to mental health last year	.22	.23	.19	.16	.77	—	6.41	0	32.71
7. Healthcare visits due to mental health last year	.35	.31	.19	.11	.62	.26	.48	0	1.78

*Note.* PHQ-9 = Patient Health Questionnaire-9; CES-D = The Center for Epidemiological Studies Depression Scale; GAD-7 = Generalized Anxiety Disorder-7; PSWQ = Penn State Worry Questionnaire.

Table S1 has more descriptive information for the prospective data.

### Concurrent and Incremental Validity

Using all eight response formats, including all four response formats for both depression and worry, yields the highest concurrent accuracies to rating scales, which approach theoretically upper limits (Table 2). Rating scales’ reliability can be seen as a theoretical upper limit (Muchinsky, 1996) for assessment accuracy, which is here measured as the mean of the item-total correlation and the test–retest reliability. The language-based assessments converge with the PHQ-9 with a correlation of .78, which is close to its reliability (*r* = .79); and converges with the CES-D with a correlation of .83, which is higher than the scales’ reliability (*r* = .78). Further, the language-based assessments converge with the GAD-7 with a correlation of .77, which is .05 lower than its reliability (*r* = .82), and converge with the PSWQ with a correlation of .74, which is .06 lower than its reliability (*r* = .80).

**Table 2:** Concurrent Validity: Comparing 10-fold Cross-Validated Pearson Correlations Based on Combined Responses with the Rating Scales’ Reliability.

Assessments	Depression		Worry	
	PHQ-9	CESD	GAD-7	PSWQ
All eight response formats	.78 (.76–1.00)	.83 (.81–1.00)	.77 (.74–1.00)	.74 (.71–1.00)
Mean reliability^1^	.79 (.74–.82)	.78 (.73–.81)	.82 (.77–.85)	.80 (.76–.84)

*Note. N* = 963. All correlations were significant at *p* < .001.

PHQ-9 = Patient Health Questionnaire-9; CES-D = The Center for Epidemiological Studies Depression Scale; GAD-7 = Generalized Anxiety Disorder-7; PSWQ = Penn State Worry Questionnaire.

1 We are taking the average of the item–total correlation and the 2-week test–retest reliability of the scales (see Table S5 for individual reliability scores). Results trained to log-transformed rating scale scores are presented in Table S7.

### Concurrent Validity of Individual and Combined Response Formats

Out of the four response formats, the select words response format yields the strongest assessment accuracy for depression (*r* = .73 for PHQ-9), followed by the write phrases (*r* = .69), the write text (*r* = .69), and the write words formats (*r* = .68; Table 3). In fact, the select format yields significantly lower errors than the write words (*t* = −5.00, *p* < .001), phrases (*t* = −22,124.11, *p* < .001), and texts (*t* = −3.70, *p* < .001). For worry, select words (*r* = .67 for GAD-7) and the write words (*r* = .67) formats performed the most accurately, followed by the write phrases (*r* = .62) and texts (*r* = .59) formats. Also, the select words format yields significantly lower errors than the write phrases (*t* = −3.61, *p* < .001) and texts (*t* = −4.37, *p* < .001) formats, but not than the write words (*t* = −.57, *p* = .566) format.

**Table 3:** Concurrent Validity: The 10-fold Cross-Validated Pearson Correlations of Single Format Model Assessments and the Observed Rating Scales.

Response format	Depression Prompt		Worry Prompt	
	PHQ-9	CES-D	GAD-7	PSWQ
Select words	.73 (.70–1.00)	.77 (.74–1.00)	.67 (.64–1.00)	.66 (.63–1.00)
Write words	.68 (.65–1.00)	.73 (.70–1.00)	.67 (.64–1.00)	.66 (.63–1.00)
Write phrases	.69 (.66–1.00)	.75 (.72–1.00)	.62 (.59–1.00)	.61 (.57–1.00)
Write text	.69 (.66–1.00)	.74 (.71–1.00)	.59 (.56–1.00)	.59 (.56–1.00)

*Note. N* = 963.

All correlations were significant at p < .001.

PHQ-9 = Patient Health Questionnaire-9 assessing depression; CES-D = The Center for Epidemiological Studies Depression Scale (CES-D); GAD-7 = Generalized Anxiety Disorder-7; PSWQ = Penn State Worry Questionnaire.

Depression prompt models for the PHQ-9 and the CES-D, and worry prompt models for the GAD-7 and the PSWQ were pre-registered for the prospective data sample, which are presented in Tables 7, 8, and 9. For results where depression responses are trained to anxiety and worry scales and vice versa, see Table S8.

Notably, the language responses tend to show a consistent specificity across all the response format predictions, so *depression language* tends to assess *depression rating scales* more accurately than the anxiety/worry scales, and *worry language* assesses the *anxiety/worry rating scales* more accurately than the depression rating scales.

### Incremental Validity: Combining Response Formats Increases the Assessment Accuracy

**Figure 1.**
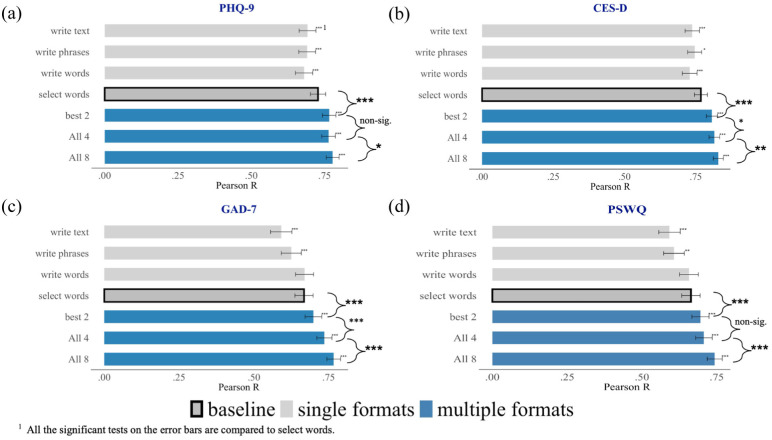
Incremental validity: The Pearson correlations of single versus combinations of response formats, (a) PHQ-9, (b) CES-D, (c) GAD-7, (d) PSWQ. *Note.* All 8 = Eight response formats. All 4 = Four response formats with the same construct prompt. Best 2 comprises select words and writes text responses from the same construct prompt, which all have the highest accuracy among the two response format combinations. The error bars show the 95% confidence intervals of each correlation. PHQ-9 = the Patient Health Questionnaire, CES-D = The Center for Epidemiological Studies Depression Scale, GAD-7 = the Generalized Anxiety Disorder-7, PSWQ = the Penn State Worry Questionnaire. The stars indicate which of the models that significantly differ in assessment error; *p < .05. **p < .01. ***p < .001. For more details, see Table S11.

Combining response formats tends to increase the assessment accuracy compared to only using one response format (Figure 1 and Table S11). For worry, combining all four formats yields the strongest correlation to the anxiety rating scales (*r* = .71–.74), whereas for depression, combining only the select words and the write text formats yields a stronger correlation to depression rating scales (*r* = .77–.81) than using all four formats (*r* = .76). Furthermore, combining both response formats and construct questions improves the assessment accuracy further, as demonstrated when using all eight response formats, which produces the highest correlations (significance testing the errors for 4 [construct congruent models, where, e.g., depression language predict depression scales] versus 8 response format yields significant differences for the PHQ-9: *t* = 2.26, *p* = .024; the CES-D: *t* = 2.73, *p* = .006; the GAD-7: *t* = 4.22, *p* < .001; and the PSWQ: *t* = 4.14, *p* < .001. Significance testing the errors for four versus the two best formats yield significant differences for the CES-D (*t* = 2.39, *p* = .017), the GAD-7 (*t* = 4.86, *p* < .001), but not for the PHQ-9 (*t* = −.98, *p* = .326) and the PSWQ (*t* = 1.24, *p* = .215). Significance testing the errors for the two most accurate formats versus the select words format yields significant differences for the PHQ-9 (*t* = 6.78, *p* < .001), the CES-D (*t* = 7.35, *p* < .001), the GAD-7 (*t* = 4.05, *p* < .001), and the PSWQ (*t* = 4.30, *p* < .001).

### Concurrent Validity Across Sample Sizes

**Figure 2.**
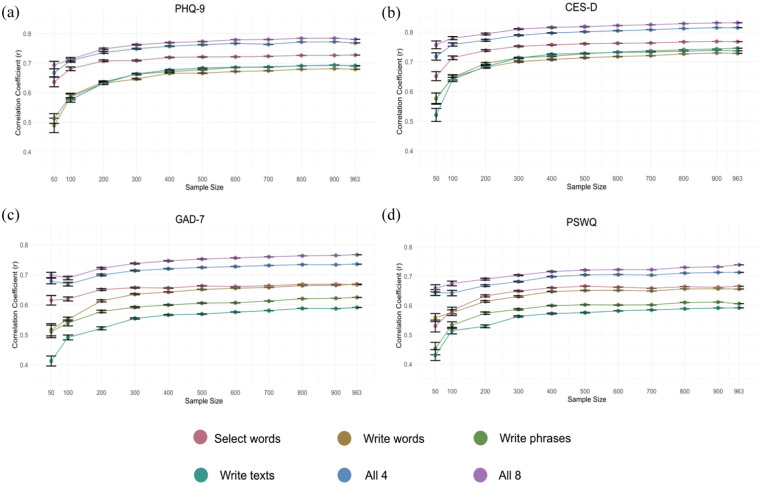
Assessment accuracy (*Pearson r*) across the number of participants included in training for each response format and the combination of 4 and 8 formats assessing (a) PHQ-9, (b) CES-D, (c) GAD-7, and (d) PSWQ. *Note.* PHQ-9 = the Patient Health Questionnaire, CES-D = The Center for Epidemiological Studies Depression Scale, GAD-7 = the Generalized Anxiety Disorder-7, PSWQ = the Penn State Worry Questionnaire.

Assessing concurrent validity across sample sizes shows how much data are necessary to achieve reliable and accurate model performance in clinical assessments. Figure 2a to d shows the concurrent accuracy achieved by models trained on different sample sizes. Overall, the models based on the select words format, including the models based on 4 and all 8 response formats, reach a high convergent accuracy at around 100 participants (although with more participants, the accuracy increases, and the error bars, of course, reduce). The writing-based formats require some more training examples than the select words format before reaching a more stable performance estimate; after 100 participants, all models are within ±0.24 of the performance estimate based on all participants; after 300 participants, all models are within ±0.09; after 500 participants, all models are within ±0.05; and after 700 participants all models are within ±0.03.

### Face Validity: Visualizations of the Language Predictive of Depression and Anxiety

**Figure 3.**
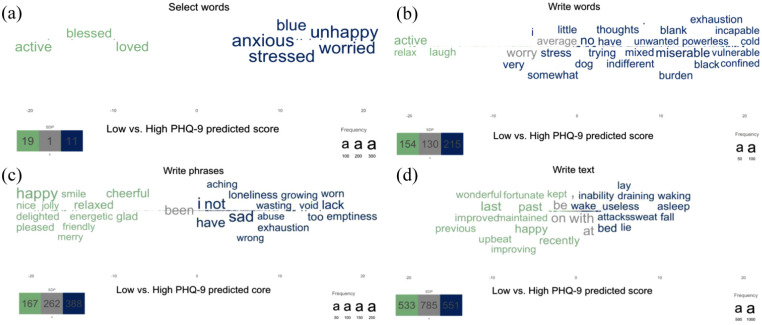
Statistically significant words related to low (green) versus high (blue) predicted PHQ-9 scores across the response formats, including (a) select words, (b) write words, (c) write phrases, and (d) write text. *Note.* The models were trained to the normalized rating scale scores. The more open the word responses are, the more statistically significant words. The open-ended response formats include more function words. Colored words are statistically significant when correcting for multiple comparisons using “False Discovery Rate.”

**Figure 4.**
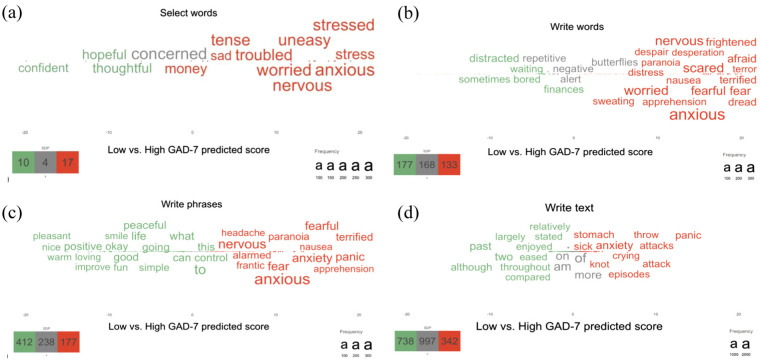
Statistically significant words related to low (green) versus high (red) predicted GAD-7 scores across the response formats, including (a) select words, (b) write words, (c) write phrases, and (d) write text. *Note.* The models were trained to the normalized rating scale scores. The more open the word responses are, the more statistically significant words there are. The open-ended response formats include more function words. Colored words are statistically significant when correcting for multiple comparisons using “False Discovery Rate.”

Plotting statistically significant word responses according to rating scales shows words related to low versus high scores (Figure 3a–d; Figure 4a–d). For all word plots, high levels of mental health are described with words such as *happy*, *glad*, and *blessed*—whereas high levels of depression tend to be described with words such as *sad* and *blue*, and worry with words such as *anxious*, *worried*, and *stress*. The select words format yields a more constrained descriptive representation (fewer statistically significant words) compared to the more open response formats. It is also noticeable that word plots based on text responses demonstrate more function words.

### Discriminant Validity

Whereas models based on several response formats tend to yield the strongest correlations to a targeted rating scale (Tables 2, 3, S11), the model prediction scores of different rating scales yield extremely strong correlations (Table 4). For example, the correlations between the predictions of the depression rating scale the PHQ-9, and the predictions of the anxiety rating scale the GAD-7, based on all eight response formats correlate with *r* = .93, whereas when based on single-response formats, the predictions only correlate with *r* = .51–.65; this may indicate poor discriminant validity. Hence, using responses from more formats when training the model increases the assessment accuracy, which may be at the cost of discriminant validity.

**Table 4.** Discriminant Validity: Correlations Between Language-Based Assessments Across Models Based on Different Response Formats.

	Language response format	Rating scale	1.	2.	3.
1.	All 8	PHQ-9	—	—	—
2.		CES-D	.97	—	—
3.		GAD-7	.93	.95	—
4.		PSWQ	.85	.87	.93
			5.	6.	7.
5.	All 4 depression	PHQ-9	—	—	—
6.		CES-D	.98	—	—
7.	All 4 worry	GAD-7	.68	.68	—
8.		PSWQ	.64	.65	.93
			9.	10.	11.
9.	Depression select words	PHQ-9	—	—	—
	10.	CES-D	.99	—	—
11.	Worry select words	GAD-7	.64	.65	—
12.		PSWQ	.62	.63	.97
			13.	14.	15.
13.	Depression write words	PHQ-9	—	—	—
14.		CES-D	.99	—	—
15.	Worry write words	GAD-7	.58	.59	—
16.		PSWQ	.58	.59	.97
			17.	18.	19.
17.	Depression write phrases	PHQ-9	—	—	—
18.		CES-D	.99	—	—
19.	Worry write phrases	GAD-7	.55	.56	—
20.		PSWQ	.55	.56	.95
			21.	22.	23.
21.	Depression write text	PHQ-9	—	—	—
22.		CES-D	.98	—	—
23.	Worry write text	GAD-7	.56	.56	—
24.		PSWQ	.51	.51	.93

*Note. N* *=* 963.

All correlations are p < .001.

PHQ-9 = Patient Health Questionnaire-9 assessing depression; CES-D = The Center for Epidemiological Studies Depression Scale (CES-D); GAD-7 = Generalized Anxiety Disorder-7; PSWQ = Penn State Worry Questionnaire.

#### Developing Models Assessing the Difference Score

Although using multiple response formats appears to yield predictions with lower discriminant validity (i.e., strong inter-correlations), it is possible to create models predicting the normalized difference score between rating scales more accurately with multiple, as compared with single response formats (Table 5; Table S3). With all eight formats, it is possible to assess the difference score of the PHQ-9 and the GAD-7 with a correlation of *r* = .38, whereas only using a single response format for one construct yields accuracies ranging from *r* = .07 to .20 (single responses in Table S3). Hence, it is possible to adapt the models according to different needs.

**Table 5.** Discriminant Validity: 10-Fold Cross-Validated Correlations Between Language-Based Assessments of Difference Scores and Observed Difference Scores of Rating Scales.

Language responses	Response format	PHQ-9−GAD-7^1^	CES-D−PSWQ^2^
Depression and worry	All	.38***	.43***
Depression		.22***	.37***
Worry		.18***	.24***
Depression and worry	Select words	.37***	.40***
	Write words	.30***	.32***
	Write phrases	.24***	.30***
	Write text	.14***	.36***

*Note. N* = 963.

* *p* < .05. **p < .01. ***p < .001.

1 Predicting the difference score of the normalized PHQ-9 minus the normalized GAD-7, where normalization was achieved by respectively subtracting the column mean from each score and dividing by the column standard deviation.

2 Predicting the difference score of the normalized CES-D minus the normalized PSWQ, in the same way as the “PHQ-9−GAD-7.” For results based on single-response formats, see Table S3.

#### Visualizing Differences Between Constructs

**Figure 5.**
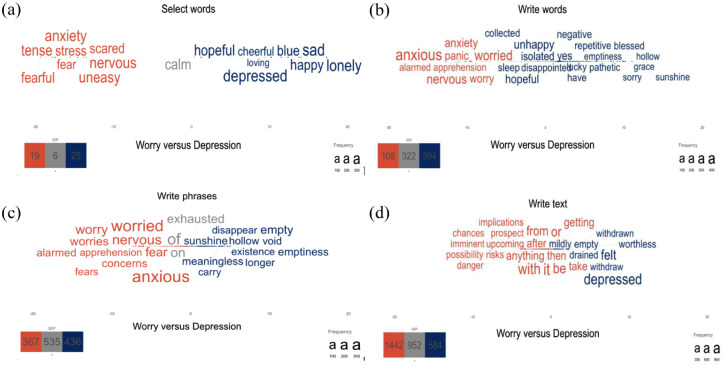
a–d | Statistically significant words related to worry (orange) versus depression (blue) responses across the response formats, including (a) select words, (b) write words, (c) write phrases, and (d) write text. *Note.* The more open the word responses are, the more statistically significant words there are. The open-ended response formats include more function words. The open-ended response formats include more function words. Colored words are statistically significant when correcting for multiple comparisons using “False Discovery Rate.”

It is also possible to differentiate the words that statistically differentiate between closely related psychological constructs (Figure 5a–d). Statistically significant words that differentiate between depression and worry responses tend to reflect the core symptoms/criteria from the DSM-V (American Psychiatric Association, 2013). Depression responses are represented by words such as *blue*, *depressed*, and *emptiness* (c.f., DSM-V criteria depressed mood), and *meaningless* and *worthless* (c.f., DSM-V criteria diminished interest or pleasure). Worry responses are represented by words such as *worried, anxious, and nervous* (c.f., DSM-V criteria excessive anxiety and worry), *panic* (c.f., DSM-V criteria difficult to control the worry).

### Prospective Sample Reliability: Pre-Registered Models Perform Well on New, Prospective Data

All of the pre-registered models performed above (*r* = .60–.79)^3^ the pre-registered cut-off (*r* = .50) on the new prospective data (Table 6); in fact, the performance tended to be slightly higher than the cross-validated estimates, where the mean difference between the prospective performance and the cross-validated estimate was 0.02 (*SD* = .03, range = −.03–.08).

**Table 6.** Prospective Sample Reliability: The Pearson Correlation of Single Response Formats From Pre-Registered Models.

Response format	Depression Prompt				Worry Prompt			
	PHQ-9		CES-D		GAD-7		PSWQ	
	Prosp.	CV	Prosp.	CV	Prosp.	CV	Prosp.	CV
Select words	.72	.73	.79	.77	.75	.67	.72	.66
Write words	.69	.68	.76	.73	.65	.67	.64	.66
Write phrases	.72	.69	.76	.75	.66	.62	.67	.61
Write text	.66	.69	.73	.74	.63	.59	.60	.59

*Note. N* = 145. CV correlations are from Table 3.

PHQ-9 = Patient Health Questionnaire-9 assessing depression; CES-D = The Center for Epidemiological Studies Depression Scale (CES-D); GAD-7 = Generalized Anxiety Disorder - 7; PSWQ = Penn State Worry Questionnaire.

For results where depression responses are trained to anxiety and worry scales and vice versa, see Table S9.

### Test–Retest Reliability

The test–retest reliability over 2 weeks was moderate to strong for the pre-registered models in the prospective dataset (Table 7). The correlation for assessing depression from depression prompts with single formats was *r* *=* .66–.75, and for assessing worry/anxiety from worry prompts was *r* = .52–.62, whereas the reliability for the rating scales was all very strong (*r* = .85–.89).

**Table 7.** Test–Retest Reliability of the Pre-Registered Models: The Pearson Correlation Between Time 1 and 2.

Response format	Depression prompt		Worry prompt	
	PHQ-9	CES-D	GAD-7	PSWQ
Select words	.69	.69	.60	.55
Write words	.74	.75	.52	.54
Write phrases	.71	.72	.62	.60
Write text	.66	.66	.53	.52
All 4^1^	.75	.76	.69	.66
All 8^1^	.76	.77	.73	.67
Rating scale	.85	.87	.86	.89

*Note. N* = 122.

PHQ-9 = Patient Health Questionnaire-9 assessing depression; CES-D = The Center for Epidemiological Studies Depression Scale (CES-D); GAD-7 = Generalized Anxiety Disorder-7; PSWQ = Penn State Worry Questionnaire.

1 These models were not pre-registered. For results where depression responses assess anxiety and worry scales and vice versa, see Table S10.

### External Validity in the Prospective Sample: Pre-Registered Model Assessments Correlate With Self-Reported Sick-Leave and Healthcare Visits

Most response formats yield assessment scores that are weakly to moderately correlated with clinically relevant external criteria related to mental health, including sick leave over the last 3 months (*r* = .18–.28) and the last year (*r* = .17–.29), as well as healthcare visits over the last year (*r* = .18–.34; Table 8). There is a similar pattern for sick leave and healthcare visits not specifically related to mental health issues (Table S4). The write text format tended to yield stronger correlations than select words, write words, and write phrases in all but one instance (although the differences tended to be non-significant); this is even though these latter formats tend to be more accurate in predicting rating scales than the write text response format. Further, language-based predictions of the rating scales based on the write text format tend to show slightly higher correlations with the external criteria than the actual observed scores (e.g., self-reported sick leave due to mental health over the last year yields a correlation of *r* = .26 with the language-based assessments of CES-D, and only a correlation of *r* = .23 with the observed CES-D). In short, among the language-based response formats, the write text format tends to yield the strongest (or on par with the strongest) correlation to the mental health-related criteria (22 of 24 cases; Table 8) and the general health-related criteria (16 of 24, Table S4). Compared to the observed rating scales, the language-based assessments exhibit an equal or stronger correlation to the externally related criteria of mental health in 9 of 12 cases (Table 8) and for general health in 11 of 12 cases (Table S4).

**Table 8.** External Validity in the Prospective Sample: The Pearson Correlation of Single Response Formats Analyzed Using Pre-Registered Models Correlate with Self-Reported External Criteria.

Response format	Sick leave due to mental health issues over the last 3 months				Sick leave due to mental health issues over the last year				Healthcare visits due to mental health issues over the last year			
	Language-based assessments				Language-based assessments				Language-based assessments			
Depression prompt	PHQ-9^LBA^	CES-D^LBA^	GAD-7^LBA^	PSWQ^LBA^	PHQ-9^LBA^	CES-D^LBA^	GAD-7^LBA^	PSWQ^LBA^	PHQ-9^LBA^	CES-D^LBA^	GAD-7^LBA^	PSWQ^LBA^
Select words	.24**	.24**	.22**	.23**	.21*	.22**	.20*	.21*	.27**	.26**	.24**	.23**
Write words	.20*	.20*	.20*	.21*	.17*	.17*	.19*	.19*	.20*	.20*	.19*	.18*
Write phrases	.23**	.23**	.21*	.18*	.14	.14	.14	.12	.26**	.26**	.22**	.20*
Write text	.24**	.28***	.28***	.24**	.22**	.26**	.29***	.25**	.32***	.34***	.32***	.29***
Worry prompt												
Select words	.12	.12	.11	.09	.13	.10	.12	.11	.12	.12	.11	.09
Write words	.11	.11	.10	.09	.15	.14	.14	.11	.11	.12	.09	.09
Write phrases	.01	.02	.02	−.01	.08	.10	.11	.07	.01	.00	−.02	−.05
Write text	.18*	.19*	.18*	.12	.17*	.17*	.17*	.11	.28***	.30***	.26**	.20*
All 8 ^(not pre-reg.)^	.24**	.26**	.19*	.12	.21*	.22*	.21*	.14	.30***	.31***	.21**	.14
	Rating Scales				Rating Scales				Rating Scales			
	PHQ-9	CES-D	GAD-7	PSWQ	PHQ-9	CES-D	GAD-7	PSWQ	PHQ-9	CES-D	GAD-7	PSWQ
	.29***	.30***	.21**	.15	.22**	.23**	.19*	.16	.35***	.31***	.19*	.11

*Note. N* = 145.

PHQ-9 = Patient Health Questionnaire-9 assessing depression; CES-D = The Center for Epidemiological Studies Depression Scale (CES-D); GAD-7 = Generalized Anxiety Disorder-7; PSWQ = Penn State Worry Questionnaire. not pre-reg. = models not being pre-registered.

* *p* < .05. **p < .01. ***p < .001.

### Information Content Across Response Formats

The response formats differ in the information content they capture (Table 9). The write phrases format yields the highest information content (Diversity Index = 561.0 for depression and 532.2 for anxiety), whereas the select words format has the lowest (Diversity Index = 23.7 for depression and 26.4 for anxiety). It is noteworthy that the information content of rating scale responses is smaller than that from the responses of open-ended formats (the write words, texts, and phrases formats) but larger than the select words format.

**Table 9.** Information Content: The Diversity Index of Responses From the Different Assessment Formats.

Response format	Diversity index^1^			
	Depression		Worry	
	Dev.	Prosp.	Dev.	Prosp.
Select words	23.7	27.2	26.4	27.3
Write words	389.4	215.7	343.1	189.0
Write phrases	561.0	312.7	532.2	337.5
Write text	437.3	326.9	460.9	342.5
Rating scales	Depression		Worry/Anxiety	
PHQ-9	60.7	59.9	—	—
CES-D	45.7	46.2	—	—
GAD-7	—	—	47.1	46.7
PSWQ	—	—	51.6	52.1

*Note. N* = 963 for the Development (Dev.) set; *N* = 145 for the Prospective (Prosp.) set.

1 The power of 2^Shannon entropy^ is used (see Kjell et al., 2024).

### Time Burden: The Time Taken to Complete Each Response Format

The survey design enabled us to measure the response time describing both constructs for each response format (Table 10). Response times with a Z-score of ±3.29 were set to the value corresponding to that Z-score.^4^ The median completion time of the select words format for both depression and worry (Median = 61 s.) was significantly smaller than the aggregated completion time of the PHQ-9 and the GAD-7 (Median = 85 s., *t* = −114.05, *p* < .001) and the CES-D and PSWQ (Median = 135 s, *t* = −36.59, *p* < .001). The second to fastest natural language response format was the write words (Median = 134 s.), followed by the write phrases (Median = 159 s.), and last, the write text format (Median = 243 s.). The response time for the response formats varies widely, where the write text format, on average, takes more than four times as long as the select words format.

**Table 10:** Time Burden: Completion Time (in Seconds) to Answer the Different Response Formats.

		Mean*		*SD*		Median	
Measure		Dev.	Prosp.	Dev.	Prosp.	Dev.	Prosp.
Depression + worry	Select words	75	62	46	30	61	55
	Write words	170	148	117	91	134	120
	Write phrases	213	167	159	104	159	133
	Write text	307	217	209	121	243	180
PHQ-9 + GAD-7		100	78	54	40	85	66
CES-D + PSWQ		156	138	77	62	135	120
PHQ-9		55	41	31	20	47	37
CES-D		79	67	40	28	68	61
GAD-7		42	35	25	21	35	29
PSWQ		74	67	40	32	64	58

*Note. N* = 963 for the Development (Dev.) set; *N* = 145 for the Prospective (Prosp.) set.

PHQ-9 = Patient Health Questionnaire-9; CES-D = The Center for Epidemiological Studies Depression Scale; GAD-7 = Generalized Anxiety Disorder-7; PSWQ = Penn State Worry Questionnaire.

Numbers are in seconds for “median” and “mean.”

* The survey program did not record the completion time of depression formats and anxiety formats separately.

## Discussion

The present study evaluated several validity and reliability aspects of language-based response formats ranging from more closed-ended to open-ended, using the SEMP design. Overall, the language-based assessments yielded high validity against self-reported rating scales and self-reported external criteria, and moderate to strong reliability, where the different response formats come with different strengths and weaknesses (Table 11). For example, in the prospective sample, the pre-registered models demonstrated high concurrent validity with depression and worry/anxiety rating scales, which all performed above the pre-registered cut-off. The select words format tended to yield the highest assessment accuracy to rating scales, closely followed by the other formats. These findings align with previous research on the utility of natural language in capturing subjective experiences (e.g., Kjell et al., 2021, 2024), emphasizing its potential to complement traditional methods assessing depression/worry in clinical settings. The results demonstrate that both simple, select-based formats and more elaborate, open-ended text responses can achieve high levels of accuracy, highlighting their potential viability for diverse clinical and research applications, ranging from rapid assessments in time-constrained settings to more detailed evaluations.

**Table 11:** Overview Summary Comparing the Best Across Response Formats.

	*r Mean (range)*							
Response formats	Predicting rating scales^1^	Discriminant validity^2^	LBA: depression—anxiety^3^	Prospective sample reliability	Test–retest reliability	External criteria	Median time (sec.)^4^	Diversity index^5^
All 8	.78 (.74–.83)	.90 (.85–.95)	.41 (.38–.43)	.78 (.72–.85)^9^	.73 (.67–.77)^10^	.21 (.12–.31)	488	646
All 4	.76 (.71–.81)	.66 (.64–.68)	.25 (.18–.37)	.77 (.74–. 83)	.71 (.66–.76)	—	244	571
2 formats^6^	.73 (.65–81)	.63 (.61–.64)	.21 (.11–.40)	—	—	—	122	434
2 constructs^7^	.71 (.65–.79)	.92 (.88–.95)	.30 (.14–.40)	—	—	—	122	208
Single formats^8^								
Select words	.66 (.56–.77)	.63 (.62–.65)	.23 (.17–.35)	.69 (.55–.79)	.62 (.55– .69)	.17 (.09–.27)	28^11^	24
Write words	.64 (.55–.73)	.58 (.58–.59)	.18 (.14–.23)	.65 (.57–.76)	.61 (.45–.75)	.15 (.09–.21)	60	389
Write phrases	.62 (.51–.75)	.56 (.55–.56)	.15 (.09–.23)	.66 (.59–.76)	.66 (.60–.72)	.11 (−.05–.26)	67	561
Write text	.61 (.50–.74)	.54 (.51–.56)	.18 (.07–.32)	.61 (.48–.73)	.57 (.52–.66)	.24 (.11–.34)	90	437

*Note.* ^1^The mean and range are based on correlations based on depression language predicting depression rating scales; and worry language predicting worry/anxiety rating scales (i.e., dark black numbers in Table 3).^2^The correlations between language-based assessments of depression and worry come from Table 4 and Table S2. Correlations of 2 formats include language from 2 responses of different constructs. ^3^Averaged correlations from Table 5 and Table S3. ^4^Averaged median completion time from Table 10 on the prospective sample. ^5^The diversity indexes of single formats are from the average of development data in Table 9. ^6^The mean of all two response format combinations from Table S11. ^7^The mean of two constructs with the same response formats from Table S11. ^8^The mean across response formats predicting corresponding rating scale from Table 3 and Table S8. ^9^The means are from Table S9. ^10^The means are from Table S10. ^11^Since we only recorded the time for answering depression and worry together, we have divided it with here two for single response formats.

Furthermore, the open-ended response formats showed incremental validity. When combined, they yielded predictive accuracies of rating scales, often approaching the rating scales’ own reliability. Combining the eight response formats typically exhibited superior performance, followed by four formats, two formats, and lastly, one response format. These findings highlight the value of integrating diverse response types to achieve a more robust assessment of these mental health constructs compared to relying on a single format. Different formats may activate distinct cognitive processes—consistent with cognitive interview theory (Willis, 2004)—and thus elicit complementary aspects of an individual’s experience. As a result, combining formats may not only improve predictive performance but also enrich the interpretability and comprehensiveness of assessments, with potential practical benefits in both research and clinical contexts.

### Challenges and Possibilities in Discriminating Constructs

Language-based assessments based on responses from *different* construct questions yield lower correlations between depression and anxiety than rating scales (language-based assessments: *r* = .51–.65, vs. rating scales: .64–.85). However, using the *same* responses (i.e., from the same construct question(s)) for assessing the two different constructs produces scores that correlate very to extremely strongly (mean *r* = .92, range = .88–.95), indicating poor discriminant validity between depression and anxiety. Hence, although the models with the same language responses as input produce the highest assessment accuracy, it appears to be at the expense of discriminant validity. This is likely due to the shared variance in the data, where using the same language to assess a construct can be compared with attempting to assess two different constructs using the same item responses from one rating scale. Hence, future language-based assessment models should minimize shared variance by assessing distinct constructs through separate questions and responses (as is done in current practices with different rating scales), while exercising caution when using the same language responses for assessing multiple constructs.

Interestingly, though, it is possible to develop models that differentiate constructs by assessing the standardized difference scores of the rating scales. Hence, research could explore whether these discriminative models can provide insights that enhance treatment planning, such as enabling the offering of more precise and tailored interventions. Improved differentiation has the potential to enable clinicians to better tailor interventions to target the underlying problems. Distinguishing between depression and anxiety is particularly important given their overlapping symptoms and co-occurrence. By identifying distinct language markers for each condition, as highlighted by Stade et al. (2023), models can improve diagnostic accuracy and guide targeted therapeutic approaches. Furthermore, distinguishing between constructs can enhance symptom monitoring in longitudinal tracking, potentially allowing for a more precise understanding of symptom changes and ensuring timely and appropriate adjustments to treatment plans. In practice, differential models could potentially be implemented as an additional metric to aid interpretation, especially in cases of symptom overlap. However, more research is also needed to examine the value of this approach in randomized controlled trials, particularly in terms of informing treatment decisions and improving patient outcomes.

### Converging With External Criteria

The pre-registered models showed external validity to self-reported sick leave and healthcare visits. A large body of research shows that depression and anxiety are related to sick leave (for a systematic reviews see Amiri et al., 2021; for a longitudinal study, see Sandin et al., 2021 and for a clinical trial see Bjørkedal et al., 2023) and healthcare visits (Cicek et al., 2022; Keller et al., 2018) providing support for the external validity of our measures. Interestingly, the write text format tended to yield the strongest correlations, which were in 9 of 12 cases actually slightly higher or equal to the correlation of observed rating scale scores. Notably, though, all three external validity measures were skewed with a median response of 0 in the current sample. This zero inflation could impact the interpretation of relationships, potentially masking the correlational strength, particularly in the non-zero portion of the sample.

### The Validity and Interpretive Power of Open-Ended Responses

The language-based assessments can describe respondents’ answers with statistically significant words across dimensions such as low to high rating scale scores. At the individual response level, the face validity of open-ended language can reveal whether a respondent has taken the task seriously (e.g., not entering random characters to fill a text box) and whether they have understood the questions (e.g., using “Yes” or “No” instead of providing descriptive answers). This capacity for nuanced evaluation is particularly advantageous compared to closed-ended formats, where insincere responses or misinterpretations of questions or instructions may be much harder to detect. The potential of evaluating the face validity of language responses could become an important aspect in clinical practice, where it can be more straightforward to identify cases in which an individual has not answered the assessment sincerely and diligently, thereby enabling timely intervention or clarification. These insights are harder to detect in closed-ended responses (although one can look for unusual response patterns).

At the group level, visualizing open-ended language responses provides a unique source for examining the face and content validity of responses. For example, in all four response formats, respondents described their depression with words such as *blue*, *depressed*, and *emptiness*, and their anxiety with words such as *nervous*, and *panic*—which is according to the DSM-V criteria (“depressed mood” and “diminished interest or pleasure” for MDD, and “excessive anxiety and worry” and “difficult to control the worry” for generalized anxiety disorder; American Psychiatric Association, 2013). Notably, the more open-ended response formats (i.e., texts, phrases, and words rather than select words) revealed more significant words. Furthermore, the write words and phrases formats tended to contain fewer function words and more content words than the text format. Depending on how important describing individuals’ responses is, these aspects may be important to consider.

Furthermore, as demonstrated in previous research (Kjell et al., 2021) and highlighted in our Supplemental Material, open-ended responses of depression and anxiety demonstrate construct validity with references to “pain,”“pains,” and “painful” (Table S13), which tend to be significantly related to higher rating scale scores. Natural language responses can reveal symptom-related content not explicitly prompted by closed-ended items. The richer insights provided by more open-ended formats (e.g., texts, phrases, and words) enhance construct validity by capturing idiosyncratic and clinically relevant expressions of distress, thus offering a more comprehensive understanding of psychological symptoms in both research and clinical settings.

### Test–Retest Reliability and Sensitivity to Change

The pre-registered models demonstrated moderate to strong test–retest reliability across 2 weeks, whereas the rating scales’ test–retest reliability was very strong. This difference highlights a trade-off between the very strong consistency of closed-ended methods and the higher flexibility of language-based approaches. Interestingly, the phrase format tended to yield both the highest test–retest reliability and the highest information content, suggesting it may offer an optimal balance between consistency and richness. Clinically, this format could be especially useful in scenarios where time is limited but nuanced symptom expression is still valuable—such as in ongoing therapy or brief intake assessments. The lower reliability of language-based assessments might be due to several reasons: First, open-ended language responses have a larger range, openness, and dimensionality than closed-ended rating scale responses (Kjell et al., 2024), which enables them to vary more. Second, the very strong test–retest reliability of rating scales might reflect a lack of sensitivity to change, whereas the moderate to strong test–retest reliability of language-based assessments potentially reflects actual changes in depression and anxiety over 2 weeks; in other words, it could be that language-based assessments may be more equipped to capture changes. While high test–retest reliability is generally desirable as it indicates consistency, there is a point at which it can signal that a mental health assessment is not sensitive enough to detect changes in a person’s condition, lacks nuance in capturing the complexity of symptoms, or both. Detecting changes in symptoms is particularly important in both research and clinical practice, especially during the course of therapy, as it informs treatment effectiveness and necessary adjustments. Future research should explore the utility of language-based assessments for repeated, longitudinal evaluations in clinical settings to determine their sensitivity and practicality in capturing meaningful symptom changes over time.

### Optimizing Sample Sizes for Model Accuracy

Understanding how model accuracy evolves with increasing training data size helps inform decisions about the minimum number of participants needed to develop robust assessments, while also showing whether current models perform as well as possible given the training sample size. Our results show clear differences in the data requirements for achieving good performance across response formats. This information can optimize resource allocation while ensuring the validity of assessment outcomes. Our results show that models based on the select words format achieve high accuracy with as few as 100 participants, while the writing-based formats require more examples to reach stable performance. As the number of participants increases beyond 500 to 700, all models begin to stabilize, with minimal further improvement in accuracy. This flattening suggests that increasing the sample size beyond this point would not substantially enhance model performance. This plateau indicates that some response formats, such as the select words format, are more efficient for training models on smaller datasets, making them particularly suitable for studies with limited resources. This insight can guide future resource allocation, allowing researchers to prioritize formats that achieve robust performance with fewer participants.

### Balancing Response Time and Information Content

The response formats vary in response time. More open response formats require more time while having the highest ecological validity (i.e., being how we normally communicate complex psychological experiences). This trade-off between brevity and richness is an important consideration for tailoring assessments to specific contexts. The use of brief assessments is increasingly favored in various research settings; for example, in extensive online surveys where participants may lack the endurance for lengthy assessments, in longitudinal studies involving repeated measures over time, and in initial screenings aimed at rapidly identifying specific characteristics or issues prior to admitting participants into a comprehensive study (e.g., see Sandy et al., 2017).

The diversity index could explain the increased accuracy of combining response formats in assessment. In general, combining response formats yields higher self-information (a higher diversity index)—and combining more response formats yields higher assessment accuracy. However, whereas the select words format yields the strongest assessment accuracy, it has the lowest diversity index. This might be explained by how the words were selected to be the most frequent answers from a previous study (Kjell et al., 2019). So, the information seems to have been optimized to capture the variance in the rating scale—but it might not be as good in other related outcomes, such as what is seen in their correlation to external criteria, including self-reported sick-leave and health visits; this requires further research. Interestingly, the phrase format tended to yield both the highest test–retest reliability and the highest information content, suggesting it may offer an optimal balance between consistency and richness. Clinically, this format could be especially useful in scenarios where time is limited but nuanced symptom expression is still valuable—such as in ongoing therapy or brief intake assessments.

### Clinical Relevance and Applications in Practice

Considering that language comprises higher range, resolution, dimensionality, openness, and information content than rating scales (Kjell et al., 2024), it has the potential to be more accurate in converging with important clinically relevant behaviors (i.e., external validity), and thus capturing more variance with signs and symptoms. Effectively getting this information extracted with standardized tools is still an ongoing effort, and particularly few works have explored different natural language response formats.

The results from our study indicate that language-based assessments can provide accurate severity scores for depression and anxiety as well as clinically meaningful descriptions. The severity scores from language-based assessments moderately to strongly converge with rating scales, and the *write text format* showed similar or even higher external validity compared to self-reports for the criterion variables. However, the write text format is also the most time-intensive. Its use may therefore be more suitable in contexts where a comprehensive understanding of the patient’s mental health is essential, such as during intake or diagnostic evaluations. Conversely, faster formats like select or write descriptive words are more practical in time-constrained settings or when frequent repeated assessments are required.

Beyond the severity scores, the open-ended response formats also comprise an individual’s unique descriptions of symptoms (e.g., *I feel overwhelmed*) and experiences (e.g., *I’m avoiding talking to others because my emotions are unpredictable and I worry that the mask might slip*). These descriptions can be presented to clinicians to support them in reaching a thorough understanding of a patient’s condition during, for example, screening, intake, or follow-up. The descriptions can also help clinicians identify specific areas or topics to explore in therapy, such as addressing expressed emotions, or concerns. By providing a detailed and personalized picture of a patient’s mental state, these insights can guide the planning of therapy sessions, ensuring that the topics addressed are tailored to the patient’s unique experiences and needs. Hence, language-based assessments can support person-centered care by providing comprehensive, person-centered insights into their mental health, aligning with the aim of prioritizing individuals’ values, needs and preferences in care planning (American Geriatrics Society Expert Panel on Person-Centered Care, 2016). Additionally, in the course of treatment, these descriptions can be shared with patients to help them reflect on and better understand the trajectory of their mental health (Folkersma et al., 2021; Morris et al., 2010). The potential therapeutic utility of this approach warrants further research (see Table 12 for suggested response formats for different clinical and research use cases).

**Table 12:** Suggested Response Formats for Different Clinical and Research Use Cases.

Use case	Recommended format(s)	Rationale	Key trade-offs
Time-constrained clinical screening	Select words	Fast to complete, high convergence with self-report	Limited descriptive richness and lower external validity
In-depth intake assessments	Combining select words and write text or phrases	Combining formats increases convergent and external validity. Open formats provide rich content for understanding patient experiences	Longer response burden, potential overlap in constructs
Research aiming for maximal predictive accuracy				
Ecologically valid and person-centered research	Write text or write phrases	Allow participants to express nuanced and unique experiences. External validity.	Lower efficiency compared to closed formats

*Note.* Formats are limited to prompted participant or patient language. Other formats for language-based assessments also exist such as passive monitoring of social media data (Eichstaedt et al., 2018). Moreover, language data can be combined with other clinically relevant information, including sensor data from devices like smartphones.

The different characteristics of the response formats further increase the clinical relevance of language-based assessments by offering different clinical applications. In clinical situations with time constraints, clinicians might prioritize faster response formats, such as the *select* or *write* descriptive words formats, where respondents complete the former faster than the corresponding rating scales. In situations where accuracy and a thorough understanding of the patients are crucial, combining response formats, such as using the *select words* and the *write text* formats. The *select words* format offers a quick and accurate option, and combining it with the *write text* format provides incremental validity and a deeper, more personal assessment, with higher chance of uncovering unique complex experiences. Furthermore, when differentiating between mental illnesses is essential, it is important to use different language questions in the assessments (like one uses different rating scales to assess depression versus anxiety) or use a model trained to yield a difference score.

Language-based assessments support and align with the digital transformation of healthcare (Warraich et al., 2018). Patients could, for example, be invited online to complete a survey where they openly describe their mental health before a meeting, which will not take time from the clinician’s work, and it can allow the clinician to prepare the session by reviewing both a severity score and a personal description of the patient’s unique experiences and symptoms. Additionally, language-based assessment can be clinically valuable in app-based interventions (Bakker et al., 2016), where patients gain insights into their longitudinal time. Such insights may empower patients to better understand their unique experiences and mental health patterns and progress, fostering engagement and self-management. Furthermore, the time spent on describing one's mental health can potentially be seen as a type of *expressive writing* intervention in itself, leading to improved health (e.g., Lepore, 1997; Lepore et al., 2002; Pennebaker & Beall, 1986; Smyth, 1998); however, to what extent this is the case for our specific open-ended measures requires future research. Future studies could investigate whether the therapeutic effects of expressive writing extend to these open-ended formats, enhancing their dual value as both assessment tools and interventions. Future research could also aim to disentangle this potential effect when evaluating therapeutic interventions, as it is important to distinguish the benefits derived specifically from the intervention itself versus those potentially arising from the act of completing the open-ended assessment.

Lastly, evaluating and visualizing the language patterns driving language-based assessment models—such as through word figures—can provide valuable insights into how AI interprets and assesses depression or anxiety severity from language. These insights can be particularly valuable in clinical settings, such as diagnostic evaluations, where understanding the linguistic markers of mental health can support clinicians in identifying key symptoms. Additionally, in research contexts, these visualizations can help elucidate the psychological mechanisms underlying these disorders, providing a deeper understanding of the relationship between language use and mental health.

### Limitations and Directions for Future Research

Language-based assessments, like rating scales, are not objective truths of psychological constructs. Like research developing stronger rating scales, our work evaluates approaches to using language-based assessments that meet more criteria of validity and reliability to get closer to latent true scores. However, more extensive evaluations are available and should be done to further establish their validity and reliability, such as their ability to converge with best-estimate assessments (e.g., Eijsbroek et al., 2024; Spitzer, 1983) as well as construct relevant observable (i.e., not only self-reported) behaviors.

Our results focus on overall accuracy metrics rather than metrics for individual preferences toward specific response formats. There also could be systematic *individual differences*, where certain individuals may express themselves better with certain response formats—or situational factors influencing the accuracy of the response formats differently. Future research could, for example, examine whether accuracy errors are related to personality traits, educational level, language skill levels, and so on and whether accuracy errors differ across different settings/contexts. Our work lays a foundation informing which response formats seem to work best on average and thus could be built on for work pursuing personalized response formats. Further, research could also systematically examine different prompts or questions to assess their influence on responses and potential differences in any demand characteristics.

The study also has methodological limitations. The questions enabling open-ended responses were presented before the list of words and closed-ended rating scales. We implemented this order to avoid the predefined word lists and items to influence respondents’ open-ended answers. However, one possibility is that the open-ended language responses may have introduced priming effects (e.g., Mohan et al., 2016) influencing how they answer the rating scale items. Hence, this priming effect may overestimate the correlation estimate. Future studies should consider randomizing the order of open-ended formats and rating scales to disentangle the effects of question order.

Moreover, since this study includes only online samples with, on average, subclinical levels of depression and anxiety severity (i.e., scores below typical clinical cut-offs) in the prospective sample, caution is advised when generalizing the findings beyond this population and context. Future studies should include diverse clinical populations to enhance the generalizability and applicability of language-based assessments in clinical settings. For instance, incorporating participants from outpatient and inpatient clinics, as well as individuals from different cultural and socioeconomic backgrounds, could provide deeper insights into the robustness and validity of these assessments. Additionally, longitudinal studies in clinical contexts could help establish their utility for monitoring treatment progress and outcomes. Further, language-based assessments need to be validated in specific populations and contexts as well as follow relevant regulations (e.g., see the AI act [Hauglid & Mahler, 2023, and Veale & Zuiderveen Borgesius, 2021], and the CE-mark in the EU [European Commission, 2023]).

Last, the current large language model was not explicitly trained for mental health assessment tasks; future research could examine whether finetuning the models may increase the assessment accuracy even further. However, fine-tuning large language models typically requires substantial amounts of data. Preliminary results from our merged dataset, encompassing over 15,000 probed language responses from multiple studies on mental health, have not yielded significant gains in accuracy. We experimented with both *domain-specific fine-tuning*—training the model on our corpus of mental health-related descriptions to improve its contextual understanding—and *task-specific fine-tuning*, where the model was directly trained to assess depression and anxiety rating scale scores. However, neither approach led to significant improvements (for a paper specifically examining fine-tuning strategies for mental health assessments using RoBERTa, see Ganesan et al., 2021). These findings, combined with the high accuracies achieved in the current study, suggest that RoBERTa-large demonstrates strong generalization capabilities to mental health language without fine-tuning it. Future research could also explore whether greater accuracy can be achieved using other large language models and different predictive model frameworks. Box 1 demonstrates R-code, exemplifying how the registered language models can be applied in future research using the *text* package (Kjell, Giorgi, & Schwartz, 2023; see Table S6 for a list of pre-registered models available for automatic download).

**Box 1:** Example Code for Using the Open Pre-Registered Models.

**# Install the text package** install.packages("text") library(text) textrpp_install() textrpp_initialize() **# Example text to access** text_to_assess <- "Most of the time, I have a hard time finding meaning in life. I feel down and blue all the time." **# This function automatically downloads the pre-registered models, transforms the text into word embeddings, and applies the model to the word embeddings** prediction <- textAssess( texts = text_to_assess, dim_name = FALSE, model_info = "depression_text_phq9_roberta23_gu2024")

## Conclusions

The results provide strong evidence demonstrating concurrent, incremental, discriminant, face, and external validity of the different response formats. Using the *Sequential Evaluation and Model Pre-registration* design, we find that the pre-registered models produce robust prospective sample reliability and test–retest reliability. We show how the response formats differ in, for example, accuracy, visualizations, time burden, and information content. The select response format exhibited superior performance across a range of metrics, including assessment accuracy and response time – however, a list of words is not the natural way of communicating complex psychological phenomena; it produces constrained visualizations and less external validity than text responses. If high accuracy matters, combining the select words and write text formats could be an option. The overall high validity and reliability across the response formats provide the possibility to choose formats according to different research and clinical needs varying in accuracy requirements, time constraints, and response openness. While these findings suggest promising directions, further work is needed to evaluate these formats and models in clinical contexts.

## Supplemental Material

sj-docx-1-asm-10.1177_10731911251364022 – Supplemental material for Natural Language Response Formats for Assessing Depression and Worry With Large Language Models: A Sequential Evaluation With Model Pre-Registration Supplemental material, sj-docx-1-asm-10.1177_10731911251364022 for Natural Language Response Formats for Assessing Depression and Worry With Large Language Models: A Sequential Evaluation With Model Pre-Registration by Zhuojun Gu, Katarina Kjell, H. Andrew Schwartz and Oscar Kjell in Assessment

## Notes

1. One participant used words like “Yes, No, No, No, No” in “word” format to respond. Another participant did not respond to “word,”“phrase,” and “text.” Both cases are included in the results, given that the number is small relative to the sample size, and the unintended responses occurred only in restricted responses.

2. The pre-registration did not include these exclusion steps, they follow previous research; for example, Kjell et al., 2016, 2022.

3. The registered models for PSWQ were first based on reversing the items incorrectly by subtracting 6 rather than 4 from the item score; we have retrained the models using the correct total scores. For transparency, the incorrect models are still open and called *model_name*_incorrect, and the newly updated models updated after the first analysis are called *model_name*_corrected. In the main document, we are reporting the results for the correct models (though note that it did not make a big difference).

4. Not adjusting for outliers still yields a significant difference: The median completion time of the select words format for both depression and worry (Median = 61 s.) was significantly smaller than the aggregated completion time of the PHQ-9 and the GAD-7 (Median = 85 s, *t* = −6.78, *p* < .001) and the CES-D and PSWQ (Median = 135 s, *t* = −19.07, *p* < .001).
