# Supplementary material for: Natural Language Response Formats for Assessing Depression and Worry With Large Language Models: A Sequential Evaluation With Model Pre-Registration
Source: Assessment. 2025 Sep 20;33(6):927–53. doi: 10.1177/10731911251364022 (PMC13379617; doi:10.1177/10731911251364022)
Supplement: sj-docx-1-asm-10.1177_10731911251364022 – Supplemental material for Natural Language Response Formats for Assessing Depression and Worry With Large Language Models: A Sequential Evaluation With Model Pre-Registration [file sj-docx-1-asm-10.1177_10731911251364022.docx]

**Supplementary Material**

| **Table S1.**  *Pearson Correlations and Descriptives of the Rating Scales* | | | | | | | | | | | | |
| --- | --- | --- | --- | --- | --- | --- | --- | --- | --- | --- | --- | --- |
| **Measure** | **1.** | **2.** | **3.** | **4** | **5.** | **6.** | **7.** | **8.** | **9.** | **Mean** | **Median** | **SD** |
| **1. PHQ-9** | - | - | - | - | - | - | - | - | - | 8.25 | 7 | 7.09 |
| **2. CES-D** | .92 | - | - | - | - | - | - | - | - | 20.16 | 18 | 14.63 |
| **3. GAD-7** | .82 | .86 | - | - | - | - | - | - | - | 7.84 | 7 | 6.43 |
| **4. PSWQ** | .65 | .70 | .76 | - | - | - | - | - | - | 37.88 | 41 | 17.33 |
| **5. Sick-leave last 3 months^1^** | .29 | .30 | .21 | .15 | - | - | - | - | - | 2.49 | 0 | 11.97 |
| **6. Sick-leave last year^1^** | .22 | .23 | .19 | .16 | .77 | - | - | - | - | 6.41 | 0 | 32.71 |
| **7. Healthcare visits last year^1^** | .35 | .31 | .19 | .11 | .62 | .26 | - | - | - | .48 | 0 | 1.78 |
| **8. Sick-leave last 3 months** | .21 | .18 | .13 | .07 | .79 | .61 | .47 | - | - | 4.15 | 0 | 14.82 |
| **9. Sick-leave last year** | .15 | .12 | .12 | .07 | .54 | .72 | .16 | .83 | - | 11.78 | 2 | 44.92 |
| **10. Healthcare visits last year** | .35 | .20 | .13 | .07 | .49 | .18 | .75 | .57 | .31 | 1.28 | 0 | 2.29 |
| Notes. *N* = 145; PHQ-9: Patient Health Questionnaire - 9; CES-D: The Center for Epidemiological Studies Depression Scale; GAD-7: Generalized Anxiety Disorder - 7; PSWQ: Penn State Worry Questionnaire. ^1^ = due to mental health. | | | | | | | | | | | | |

| **Table S2.**  *Discriminative Validity: Correlations Between Language-Based Assessments Across Response Formats* | | | |
| --- | --- | --- | --- |
| ***Language response format – Rating Scale*** |  |  |  |
|  | ***1.*** | ***2.*** | ***3*** |
| ***1. 2 depression response formats - PHQ-9 in average*** | *-* | *-* | *-* |
| ***2. 2 depression response formats - CES-D in average*** | .99 | - | - |
| ***3. 2 worry response formats - GAD-7 in average*** | .63 | .64 | - |
| ***4. 2 worry response formats - PSWQ in average*** | .61 | .62 | .95 |
|  | ***5.*** | ***6.*** | ***7.*** |
| ***5. 1 format + 2 constructs - PHQ-9 in average*** | *-* | *-* | *-* |
| ***6. 1 format + 2 constructs - CES-D in average*** | .98 | - | - |
| ***7. 1 format + 2 constructs - GAD-7 in average*** | .94 | .95 | - |
| ***8. 1 format + 2 constructs - PSWQ in average*** | .88 | .90 | .94 |
| Notes: *N* = 963.  PHQ-9: Patient Health Questionnaire - 9; CES-D: The Center for Epidemiological Studies Depression Scale; GAD-7: Generalized Anxiety Disorder - 7; PSWQ: Penn State Worry Questionnaire. | | | |

| **Table S3.**  *The Discriminant Validity: Correlations between Language Based Assessments of Difference Scores and Rating Scale Difference Scores* | | | |
| --- | --- | --- | --- |
| **Language responses** | **Response format** | **PHQ-9 – GAD-7^1^** | **CES-D – PSWQ^2^** |
| **Depression** | **select + words** | .22 | .33 |
|  | **select + phrases** | .19 | .33 |
|  | **select + text** | .21 | .40 |
|  | **words + phrases** | .16 | .23 |
|  | **words + text** | .17 | .29 |
|  | **phrases + text** | .15 | .30 |
| **Worry** | **select + words** | .17 | .19 |
|  | **select + phrases** | .19 | .18 |
|  | **select + text** | .16 | .24 |
|  | **words + phrases** | .16 | .11 |
|  | **words + text** | .15 | .23 |
|  | **phrases + text** | .13 | .20 |
| **Depression** | **Select words** | .20**^***^** | .35**^***^** |
|  | **Write words** | .17**^***^** | .23**^***^** |
|  | **Write phrases** | .14**^***^** | .23**^***^** |
|  | **Write text** | .14**^***^** | .32**^***^** |
| **Worry** | **Select words** | .17**^***^** | .19**^***^** |
|  | **Write words** | .16**^***^** | .14**^***^** |
|  | **Write phrases** | .14**^***^** | .09**^**^** |
|  | **Write text** | .07**^*^** | .20**^***^** |
| Notes. *N* = 963.  ^1^ Predicting the difference score of the normalized PHQ-9 minus the normalized GAD-7, where normalization was achieved by respectively subtracting the column mean from each score and dividing by the column standard deviation.  ^2^ Predicting the difference score of the normalized CESD minus the normalized PSWQ. Same as “PHQ-9 - GAD-7”.  All models of 2 response formats are significantly at *p* < .001 | | | |

| **Table S4.**  *Prospective Validity to External Criteria: The Pearson Correlation of Single Responses Formats Analysed Using Pre-Registered Models Correlate with Self-Reported External Criteria* | | | | | | | | | | | | |
| --- | --- | --- | --- | --- | --- | --- | --- | --- | --- | --- | --- | --- |
|  | **Sick leave over the last 3 months** | | | | **Sick leave over the last year** | | | | **Healthcare visits over the last year** | | | |
|  | **Language-based assessments** | | | | **Language-based assessments** | | | | **Language-based assessments** | | | |
| **Depression Prompt** | **PHQ-9^LBA^** | **CES-D^LBA^** | **GAD-7^LBA^** | **PSWQ^LBA^** | **PHQ-9^LBA^** | **CES-D^LBA^** | **GAD-7^LBA^** | **PSWQ^LBA^** | **PHQ-9^LBA^** | **CES-D^LBA^** | **GAD-7^LBA^** | **PSWQ^LBA^** |
| **Select words** | .18* | .18* | .18* | .19* | .15 | .16 | .16 | .18* | .18* | .17* | .16 | .18* |
| **Write words** | .10 | .11 | .13 | .14 | .06 | .07 | .10 | .11 | .16 | .16* | .17* | .16 |
| **Write phrases** | .19* | .19* | .17* | .13 | .11 | .11 | .11 | .08 | .25** | .25** | .22** | .20* |
| **Write text** | .24** | .27** | .27*** | .24** | .21* | .23** | .25** | .21* | .23** | .24** | .21** | .19* |
| **Worry Prompt** |  |  |  |  |  |  |  |  |  |  |  |  |
| **Select words** | .07 | .07 | .07 | .07 | .08 | .07 | .08 | .09 | .09 | .10 | .09 | .08 |
| **Write words** | .07 | .07 | .06 | .05 | .10 | .09 | .09 | .06 | .07 | .06 | .04 | .03 |
| **Write phrases** | .03 | .05 | .04 | .01 | .08 | .11 | .12 | .08 | .03 | .05 | .03 | .00 |
| **Write text** | .16 | .16 | .16 | .12 | .14 | .13 | .13 | .10 | .21** | .21** | .19* | .18* |
| **8 formats^(not pre-reg.)^** | .17* | .20* | .16 | .09 | .14 | .16 | .17* | .11 | .22** | .24** | .16 | .12 |
|  | **Rating Scales** | | | | **Rating Scales** | | | | **Rating Scales** | | | |
|  | **PHQ-9** | **CES-D** | **GAD-7** | **PSWQ** | **PHQ-9** | **CES-D** | **GAD-7** | **PSWQ** | **PHQ-9** | **CES-D** | **GAD-7** | **PSWQ** |
|  | .21** | .18** | .13 | .07 | .15 | .12 | .12 | .07 | **.**25** | .20* | .13 | .07 |
| Notes. *N = 145.* All models use an embedding from Roberta large layer 23. PHQ-9 = Patient Health Questionnaire-9 assessing depression; CES-D = The Center for Epidemiological Studies Depression Scale; GAD-7 = Generalized Anxiety Disorder - 7; PSWQ = Penn State Worry Questionnaire. not pre-reg. = models not being pre-registered. *^***^ = p < .001, ^**^ = p < .01, ^*^ = p < .05* | | | | | | | | | | | | |

| **Table S5.**  *Concurrent Validity: Comparing Cross-Validated Pearson Correlations Based on Combined Responses Analyzed with Large Language Models to the Reliability of the Rating Scales.* | | | | |
| --- | --- | --- | --- | --- |
| **Response format** | **Depression** | | **Worry** | |
|  | **PHQ-9** | **CESD** | **GAD-7** | **PSWQ** |
| **All 8** | .78  (.76 - 1.00) | .83  (.81 - 1.00) | .77  (.74 - 1.00) | .74  (.71 - 1.00) |
| **Reliability measures** | | | | |
| **Average reliability^1^** | .79  (.74 - .82) | .78  (.73 - .81) | .82  (.77 - .85) | .80  (.76 - .84) |
| item-total correlation | .72  (.69 - .75) | .68  (.64 - .71) | .77  (.74 - .80) | .71  (.67 - .75) |
| test-retest reliability | .85  (.79 - .89) | .87  (.82 - .91) | .86  (.80 - .90) | .89  (.84 - .92) |
| Notes. *N* = 963. All correlations were significant at *p* < .001. PHQ-9: Patient Health Questionnaire - 9; CES-D: Center for Epidemiological Survey Depression scale for depression; GAD-7: Generalized Anxiety Disorder - 7; PSWQ: Penn State Worry Questionnaire. **^ln^** = log_e_  ^1^ Reliability is seen as the upper bound benchmark in Kjell et al., 2022; here, we are taking the average of the item-total correlation and the two-week test-retest reliability of the scales. | | | | |

| **Table S6.** *List of Pre-Registered Open Prediction Model Files* | |
| --- | --- |
| **File Name** | **Description** |
| **PHQ-9 prediction** | |
| [depressionselect_robertaL23_phq9_Gu2024.rds](https://github.com/theharmonylab/open_models/raw/main/response_format_2024/depressionselect_robertaL23_phq9_Gu2024.rds) | The format of selecting depression words predicts PHQ-9. |
| [depressionword_robertaL23_phq9_Gu2024.rds](https://github.com/theharmonylab/open_models/raw/main/response_format_2024/depressionword_robertaL23_phq9_Gu2024.rds) | Format of writing depression words predicts PHQ-9. |
| [depressionphrase_robertaL23_phq9_Gu2024.rds](https://github.com/theharmonylab/open_models/raw/main/response_format_2024/depressionphrase_robertaL23_phq9_Gu2024.rds) | The format of writing depression phrases predicts PHQ-9. |
| [depressiontext_robertaL23_phq9_Gu2024.rds](https://github.com/theharmonylab/open_models/raw/main/response_format_2024/depressiontext_robertaL23_phq9_Gu2024.rds) | The format of writing depression texts predicts PHQ-9. |
| **CES-D prediction** | |
| [depressionselect_robertaL23_cesd_Gu2024.rds](https://github.com/theharmonylab/open_models/raw/main/response_format_2024/depressionselect_robertaL23_cesd_Gu2024.rds) | The format of selecting depression words predicts CES-D. |
| [depressionword_robertaL23_cesd_Gu2024.rds](https://github.com/theharmonylab/open_models/raw/main/response_format_2024/depressionword_robertaL23_cesd_Gu2024.rds) | Format of writing depression words predicts CES-D. |
| [depressionphrase_robertaL23_cesd_Gu2024.rds](https://github.com/theharmonylab/open_models/raw/main/response_format_2024/depressionphrase_robertaL23_cesd_Gu2024.rds) | The format of writing depression phrases predicts CES-D. |
| [depressiontext_robertaL23_cesd_Gu2024.rds](https://github.com/theharmonylab/open_models/raw/main/response_format_2024/depressiontext_robertaL23_cesd_Gu2024.rds) | Format of writing depression texts predicts CES-D. |
| **GAD-7 prediction** | |
| [worryselect_robertaL23_gad7_Gu2024.rds](https://github.com/theharmonylab/open_models/raw/main/response_format_2024/worryselect_robertaL23_gad7_Gu2024.rds) | The format of selecting depression words predicts GAD-7. |
| [worryword_robertaL23_gad7_Gu2024.rds](https://github.com/theharmonylab/open_models/raw/main/response_format_2024/worryword_robertaL23_gad7_Gu2024.rds) | Format of writing depression words predicts GAD-7. |
| [worryphrase_robertaL23_gad7_Gu2024.rds](https://github.com/theharmonylab/open_models/raw/main/response_format_2024/worryphrase_robertaL23_gad7_Gu2024.rds) | The format of writing depression phrases predicts GAD-7. |
| [worrytext_robertaL23_gad7_Gu2024.rds](https://github.com/theharmonylab/open_models/raw/main/response_format_2024/worrytext_robertaL23_gad7_Gu2024.rds) | Format of writing depression texts predicts GAD-7. |
| **PSWQ prediction** | |
| [worryselect_robertaL23_pswq_Gu2024.rds](https://github.com/theharmonylab/open_models/raw/main/response_format_2024/worryselect_robertaL23_pswq_Gu2024_corrected.rds) | The format of selecting depression words predicts PSWQ. |
| [worryword_robertaL23_pswq_Gu2024.rds](https://github.com/theharmonylab/open_models/raw/main/response_format_2024/worryword_robertaL23_pswq_Gu2024_corrected.rds) | Format of writing depression words predicts PSWQ. |
| [worryphrase_robertaL23_pswq_Gu2024.rds](https://github.com/theharmonylab/open_models/raw/main/response_format_2024/worryphrase_robertaL23_pswq_Gu2024_corrected.rds) | The format of writing depression phrases predicts PSWQ. |
| [worrytext_robertaL23_pswq_Gu2024.rds](https://github.com/theharmonylab/open_models/raw/main/response_format_2024/worrytext_robertaL23_pswq_Gu2024_corrected.rds) | The format of writing depression texts predicts PSWQ. |
| Notes.  PHQ-9: Patient Health Questionnaire-9; CES-D: The Center for Epidemiological Studies Depression Scale; GAD-7: Generalized Anxiety Disorder - 7; PSWQ: Penn State Worry Questionnaire. | |

| **Table S7**  *Concurrent Validity: Comparing 10-fold Cross-Validated Pearson Correlations Based on Combined Responses with the Rating Scales’ Reliability.* | | | | |
| --- | --- | --- | --- | --- |
|  | **Depression** | | **Worry** | |
|  | **PHQ-9^ln^** | **CESD^ln^** | **GAD^ln^** | **PSWQ^ln^** |
| **All 8** | .81  (.80 - 1.00) | .82  (.81 - 1.00) | .83  (.81 - 1.00) | .71  (.69 - 1.00) |
| Notes. *N* = 963. All correlations were significant at *p* < .001. PHQ-9: Patient Health Questionnaire - 9; CES-D: The Center for Epidemiological Studies Depression Scale; GAD-7: Generalized Anxiety Disorder - 7; PSWQ: Penn State Worry Questionnaire. **^ln^** = log_e_ | | | | |

| **Table S8**.  *Concurrent Validity: The 10-fold Cross-Validated Pearson Correlations of Single Format Model Predictions and the Observed Rating Scales* | | | | |
| --- | --- | --- | --- | --- |
| **Response format** | **Worry Prompt** | | **Depression Prompt** | |
|  | **PHQ-9** | **CES-D** | **GAD-7** | **PSWQ** |
| **Select words** | .56  (.53 - 1.00) | .64  (.61 - 1.00) | .64  (.61 - 1.00) | .58  (.54 - 1.00) |
| **Write words** | .57  (.53 - 1.00) | .63  (.59 - 1.00) | .59  (.55 - 1.00) | .55  (.52 - 1.00) |
| **Write phrases** | .51  (.47 - 1.00) | .58  (.54 - 1.00) | .60  (.57 - 1.00) | .57  (.53 - 1.00) |
| **Write text** | .50  (.46 - 1.00) | .58  (.54 - 1.00) | .61  (.58 - 1.00) | .58  (.53 - 1.00) |
| Notes. *N = 963.* All correlations were significant at *p* < .001.  PHQ-9 = Patient Health Questionnaire-9 assessing depression; CES-D = The Center for Epidemiological Studies Depression Scale (CES-D); GAD-7 = Generalized Anxiety Disorder - 7; PSWQ = Penn State Worry Questionnaire. Black font = input-output congruent models where, e.g., depression responses assess depression rating scales; gray font = input-output incongruent models where depression responses assess worry/anxiety rating scales.  Depression prompt models for the PHQ-9 and the CES-D and worry prompt models for the GAD-7 and the PSWQ were pre-registered for the prospective data sample, which are presented in Tables 7, 8, and 9 (see Table SM6 for more results). | | | | |

| **Table S9**.  *Prospective Sample Reliability: The Pearson Correlation of Single Responses Formats From Pre-Registered Models* | | | | | | | | |
| --- | --- | --- | --- | --- | --- | --- | --- | --- |
| **Response format** | **Worry Prompt** | | | | **Depression Prompt** | | | |
|  | **PHQ-9** | | **CES-D** | | **GAD-7** | | **PSWQ** | |
|  | **Prosp.** | **CV** | **Prosp.** | **CV** | **Prosp.** | **CV** | **Prosp.** | **CV** |
| **Select words** | .63 | .56 | .70 | .64 | .67 | .64 | .55 | .59 |
| **Write words** | .63 | .57 | .62 | .63 | .66 | .59 | .57 | .55 |
| **Write phrases** | .59 | .51 | .61 | .58 | .65 | .60 | .60 | .57 |
| **Write text** | .62 | .50 | .65 | .58 | .54 | .61 | .48 | .57 |
| **All 4 dep** | .75 | .76 | .83 | .81 | / | | | |
| **All 4 wor** | / | | | | .76 | .74 | .74 | .71 |
| **All 8** | .77 | .78 | .85 | .83 | .78 | .77 | .72 | .74 |
| Notes. *N = 145.* CV correlations are from Table S8.  PHQ-9 = Patient Health Questionnaire-9 assessing depression; CES-D = The Center for Epidemiological Studies Depression Scale (CES-D); GAD-7 = Generalized Anxiety Disorder - 7; PSWQ = Penn State Worry Questionnaire. | | | | | | | | |

| **Table S10**.  *Test-Retest Reliability of the Pre-registered Models: The Pearson Correlation between Time 1 and 2 of predictions* | | | | |
| --- | --- | --- | --- | --- |
| **Response format** | **Worry Prompt** | | **Depression Prompt** | |
|  | **PHQ-9** | **CES-D** | **GAD-7** | **PSWQ** |
| **Select words** | .59 | .59 | .65 | .60 |
| **Write words** | .45 | .48 | .72 | .71 |
| **Write phrases** | .61 | .60 | .72 | .70 |
| **Write text** | .52 | .52 | .62 | .56 |
| **All 4^1^** | .67 | .68 | .72 | .72 |
| Notes. *N* = 122.  PHQ-9 = Patient Health Questionnaire-9 assessing depression; CES-D = The Center for Epidemiological Studies Depression Scale (CES-D); GAD-7 = Generalized Anxiety Disorder-7; PSWQ = Penn State Worry Questionnaire  **^1^** = These models were not pre-registered gray font = input-output incongruent models where depression responses assess worry/anxiety rating scales. | | | | |

| **Table S11.**  *Incremental Validity: The Pearson Correlations of Combinations of Response Formats* | | | | | |
| --- | --- | --- | --- | --- | --- |
| **Language response format** | | ***Depression scales*** | | ***Anxiety/worry scales*** | |
|  |  | ***PHQ-9*** | ***CES-D*** | ***GAD-7*** | ***PSWQ*** |
| **All 4** | | .76 | .81 | .74 | .71 |
| **Two-response formats** | |  |  |  |  |
| **select +** | **words** | .74 | .79 | .71 | .69 |
|  | **phrases** | .75 | .80 | .70 | .69 |
|  | **text** | .77 | .81 | .70 | .70 |
|  | **select^dc^** | .74 | .79 | .71 | .69 |
| **words +** | **phrases** | .70 | .76 | .70 | .67 |
|  | **text** | .73 | .78 | .70 | .69 |
|  | **words^dc^** | .72 | .77 | .70 | .68 |
| **phrases +** | **text** | .74 | .79 | .67 | .65 |
|  | **phrases^dc^** | .71 | .77 | .69 | .66 |
| **text +** | **text^dc^** | *.69* | .75 | *.66* | .65 |
| Notes. *N* = 963. **^dc^** = where the same response formats are used, we combine answers from different constructs, otherwise, depression scales are predicted using depression responses, and vice versa for anxiety/worry scales. PHQ-9 = Patient Health Questionnaire-9 assessing depression; CES-D = The Center for Epidemiological Studies Depression Scale (CES-D); GAD-7 = Generalized Anxiety Disorder - 7; PSWQ = Penn State Worry Questionnaire. | | | | | |

| **Table S12**.  *Prospective Sample Reliability: The Pearson Correlation of Single Responses Formats From Pre-Registered Models* | | | | | | | | |
| --- | --- | --- | --- | --- | --- | --- | --- | --- |
|  | **PHQ-9** | | | | **CES-D** | | | |
| **Response format** | ***r*** | ***rho*** | **MAE** | **RMSE** | ***r*** | ***rho*** | **MAE** | **RMSE** |
| **Depression Prompt** | | | | | | | |  |
| **Select words** | .72 | .75 | 3.91 | 5.09 | .79 | .79 | 7.10 | 9.17 |
| **Write words** | .69 | .70 | 4.34 | 5.40 | .76 | .76 | 8.15 | 10.10 |
| **Write phrases** | .72 | .75 | 4.26 | 5.28 | .76 | .78 | 8.40 | 10.29 |
| **Write text** | .66 | .69 | 4.57 | 5.60 | .73 | .76 | 8.58 | 10.62 |
| **Worry Prompt** | **GAD-7** | | | | **PSWQ** | | | |
| **Select words** | .63 | .78 | 3.59 | 4.50 | .70 | .68 | 10.00 | 12.32 |
| **Write words** | .63 | .68 | 4.12 | 5.06 | .62 | .64 | 10.76 | 13.51 |
| **Write phrases** | .59 | .69 | 4.34 | 5.04 | .61 | .61 | 10.82 | 13.34 |
| **Write text** | .62 | .67 | 4.58 | 5.44 | .65 | .53 | 11.39 | 14.56 |
| Notes. *N = 145.*  *r*: Pearson’s *r; rho*: Spearman’s *rho*; MAE: Mean Average Error: Mean Absolute Error; RMSE: Root Mean Squared Error;  PHQ-9 = Patient Health Questionnaire-9 assessing depression; CES-D = The Center for Epidemiological Studies Depression Scale (CES-D); GAD-7 = Generalized Anxiety Disorder - 7; PSWQ = Penn State Worry Questionnaire. | | | | | | | | |

| **Table S13.**  *Pain, pains and painful are are related to high depression and anxiety.* | | | |
| --- | --- | --- | --- |
| **Response formats** | **Dot product projection values related to high depression/anxiety** | | |
|  | **“pain”** | **“pains”** | **“painful”** |
| **Depression prompt** | | | |
| **Write words** | 8.91 | - | 10.11 |
| **Write phrases** | 3.17 | - | - |
| **Write text** | 1.50 | - | 1.68 |
| **Worry prompt** | | | |
| **Write words** | 3.60 | - | 5.05 |
| **Write phrases** | 2.49^**^ | 7.47 | - |
| **Write text** | 1.50 | 2.51 | 1.42 |
| Notes. All values are at the level of *p* < .001 except one with stars. ** = *p* < .01, * = *p* < .05. For more details about the analyses Figure 3 and 4. | | | |

**Appendix SA1: Screening questions**

**Screening question**

Indicate below whether you have been diagnosed with a psychiatric disorder **that is still/currently ongoing**. Also indicate whether this disorder is Major Depressive Disorder, Generalized Anxiety Disorder, and/or with another psychiatric disorder/illness/condition. By ongoing, we mean that the disorder described your mental state over the last two weeks.

Please select the alternatives that reflect your situation best:

- I have not been diagnosed with a psychiatric disorder/illness/condition that is currently ongoing.
- I have been diagnosed with Major Depressive Disorder (MDD) AND it is currently ongoing.
- I have been diagnosed with Generalized Anxiety Disorder (GAD) AND it is currently ongoing.
- Other: _________________

**Appendix SA2: Instructions on response formats used in depression and anxiety**

**1. Question and instructions for the depression select response item**

“**Over the last 2 weeks, have you been depressed or not?**

Please answer the question by selecting 5 descriptive words below that indicate whether you have been depressed or not. Try to weigh the strength and the number of words that describe if you have been depressed or not so that they reflect your overall personal state of depression. For example, if you have been depressed, then select more and stronger words describing this, and if you have not been depressed, then select more and stronger words describing that.

Select descriptive words relating to those aspects that are most important and meaningful to you.

Select only 5 descriptive words.”

**2. Instruction for depression word response**

“**Over the last 2 weeks, have you been depressed or not?**

Please answer the question by typing 5 descriptive words below that indicate whether you have been depressed or not. Try to weigh the strength and the number of words that describe if you have been depressed or not so that they reflect your overall personal state of depression. For example, if you have been depressed, then write more and stronger words describing this, and if you have not been depressed, then write more and stronger words describing that. Write descriptive words relating to those aspects that are most important and meaningful to you. Write only one descriptive word in each box.”

**3. Instruction for depression phrase response**

“**Over the last 2 weeks, have you been depressed or not?**

Please answer the question by typing 5 descriptive words or phrases below that indicate whether you have been depressed or not. Try to weigh the strength and the number of words or phrases that describe if you have been depressed or not so that they reflect your overall personal state of depression. For example, if you have been depressed, then write more and stronger words or phrases describing this, and if you have not been depressed, then write more and stronger words or phrases describing that. Write descriptive words or phrases relating to those aspects that are most important and meaningful to you. Write one to five words in each box.”

**4. Instruction for depression text response**

“**Over the last 2 weeks, have you been depressed or not?**

Please answer the question by typing at least a paragraph below that indicates whether you have been depressed or not. Try to weigh the strength and the number of aspects that describe if you have been depressed or not so that they reflect your overall personal state of depression. For example, if you have been depressed, then write more about aspects describing this, and if you have not been depressed, then write more about aspects describing that. Write about those aspects that are most important and meaningful to you. Write at least one paragraph in the box.”

**5. Instruction for anxiety select response**

“**Over the last 2 weeks, have you been worried or not?**

Please answer the question by selecting 5 descriptive words below that indicate whether you have been worried or not. Try to weigh the strength and the number of words that describe if you have been worried or not so that they reflect your overall personal state of worry. For example, if you have been worried, then select more and stronger words describing this, and if you have not been worried, then select more and stronger words describing that. Select descriptive words relating to those aspects that are most important and meaningful to you. Select only 5 descriptive words.”

**6. Instruction for anxiety word response**

“**Over the last 2 weeks, have you been worried or not?**

Please answer the question by typing 5 descriptive words below that indicate whether you have been worried or not. Try to weigh the strength and the number of words that describe if you have been worried or not so that they reflect your overall personal state of worry. For example, if you have been worried, then write more and stronger words describing this, and if you have not been worried, then write more and stronger words describing that. Write descriptive words relating to those aspects that are most important and meaningful to you. Write only one descriptive word in each box.”

**7. Instruction for anxiety phrase response**

“**Over the last 2 weeks, have you been worried or not?**

Please answer the question by typing 5 descriptive words below that indicate whether you have been worried or not. Try to weigh the strength and the number of words that describe if you have been worried or not so that they reflect your overall personal state of worry. For example, if you have been worried, then write more and stronger words describing this, and if you have not been worried, then write more and stronger words describing that. Write descriptive words relating to those aspects that are most important and meaningful to you. Write only one descriptive word in each box.”

**8. Instruction for anxiety text response**

“**Over the last 2 weeks, have you been worried or not?**

Please answer the question by typing at least a paragraph below that indicates whether you have been worried or not. Try to weigh the strength and the number of aspects that describe if you have been worried or not so that they reflect your overall personal state of worry. For example, if you have been worried, then write more about aspects describing this, and if you have not been worried, then write more about aspects describing that. Write about those aspects that are most important and meaningful to you. Write at least one paragraph in the box.”

**Appendix SA3: Words used in the Word Selection Response Format**

Depression words and worry words used in the word selection response format included:

| **Depression words** | | **Worry words** | |
| --- | --- | --- | --- |
| happy | blue | anxious | hopeful |
| sad | lonely | calm | sad |
| content | relaxed | happy | satisfied |
| joyful | pleased | nervous | stress |
| satisfied | stressed | relaxed | money |
| tired | depressed | concerned | anxiety |
| peaceful | active | worried | fear |
| cheerful | angry | content | tired |
| excited | fun | stressed | unworried |
| calm | loving | peaceful | troubled |
| hopeful | upbeat | upset | confident |
| down | blessed | tense | thoughtful |
| anxious | joy | scared | bothered |
| unhappy | love | carefree | peace |
| worried | loved | uneasy | untroubled |
| optimistic |  | fearful |  |

**Appendix SA4: Control items in PHQ-9 and GAD-7**

Control item of PHQ-9

| Item | Not at all | Several days | More than half the days | Nearly every day |
| --- | --- | --- | --- | --- |
| On this attention check, answer alternative 3, "More than half the days" | 〇 | 〇 | 〇 | 〇 |

Control item of GAD-7

| Item | Not at all | Several days | More than half the days | Nearly every day |
| --- | --- | --- | --- | --- |
| On this attention check, answer alternative 2, "Several days" | 〇 | 〇 | 〇 | 〇 |

**Appendix SA5: R-packages used in the analyses**

Bird, S. (2006, July). NLTK: the natural language toolkit. In *Proceedings of the COLING/ACL 2006 Interactive Presentation Sessions* (pp. 69-72).

Gagolewski, M. (2022). stringi: Fast and portable character string processing in R. *Journal of Statistical Software*, *103*, 1-59.

Lemenkova, P. (2020). R Libraries {dendextend} and {magrittr} and clustering package scipy. cluster of Python for modelling diagrams of dendrogram trees. *Carpathian Journal of Electronic and Computer Engineering*, *13*(1), 5-12.

Revelle, W., & Revelle, M. W. (2015). Package ‘psych’. *The comprehensive R archive network*, *337*(338), 161-165.

Ushey K, Allaire J, Tang Y (2024). *reticulate: Interface to 'Python'*. R package version 1.39.0, https://github.com/rstudio/reticulate, <https://rstudio.github.io/reticulate/>

Wickham, H., Averick, M., Bryan, J., Chang, W., McGowan, L. D. A., François, R., ... & Yutani, H. (2019). Welcome to the Tidyverse. *Journal of open source software*, *4*(43), 1686.
